# Clinical examination findings as prognostic factors in low back pain: a systematic review of the literature

**DOI:** 10.1186/s12998-015-0054-y

**Published:** 2015-03-23

**Authors:** Lisbeth Hartvigsen, Alice Kongsted, Lise Hestbaek

**Affiliations:** Department of Sports Science and Clinical Biomechanics, University of Southern Denmark, Odense, Denmark; Nordic Institute of Chiropractic and Clinical Biomechanics, Odense, Denmark

## Abstract

**Background:**

There is a strong tradition of performing a clinical examination of low back pain (LBP) patients and this is generally recommended in guidelines. However, establishing a pathoanatomic diagnosis does not seem possible in most LBP patients and clinical tests may potentially be more relevant as prognostic factors. The aim of this review of the literature was to systematically assess the association between low-tech clinical tests commonly used in adult patients with acute, recurrent or chronic LBP and short- and long-term outcome.

**Methods:**

MEDLINE, Embase, and MANTIS were searched from inception to June 2012. Prospective clinical studies of adult patients with LBP with or without leg pain and/or signs of nerve root involvement or spinal stenosis, receiving non-surgical or no treatment, which investigated the association between low-tech clinical tests and outcome were included. Study selection, data extraction and appraisal of study quality were performed independently by two reviewers.

**Results:**

A total of 5,332 citations were retrieved and screened for eligibility, 342 articles were assessed as full text and 49 met the inclusion criteria. Due to clinical and statistical heterogeneity, qualitative synthesis rather than meta-analysis was performed. Associations between clinical tests and outcomes were often inconsistent between studies. In more than one third of the tests, there was no evidence of the tests being associated with outcome. Only two clinical tests demonstrated a consistent association with at least one of the outcomes: centralization and non-organic signs.

**Conclusions:**

For most clinical tests in LBP there is not consistent evidence for an association with outcome. Centralization and non-organic signs are exceptions from that. None of the other clinical tests have been investigated in confirmatory studies and study quality is generally low. There is a need for hypothesis testing studies designed specifically to investigate the prognostic value of the clinical tests, and a need for standardization of the performance and interpretation of tests.

**Electronic supplementary material:**

The online version of this article (doi:10.1186/s12998-015-0054-y) contains supplementary material, which is available to authorized users.

## Background

Low back pain (LBP) is a leading cause of disability worldwide, contributing to approximately 10% of all years lived with disability [1]. It is estimated that 632 million people are affected worldwide [1] and 12-33% of adults have LBP at any given time [2]. For some, acute episodes of pain subside within days or weeks but many experience more persistent pain and recurrences are common. About two-thirds of patients presenting in primary care still report pain up to one year later or will have experienced a recurrence of pain [3,4]. Thus, LBP is to be viewed as an episodic and recurrent condition probably manifesting itself over the entire lifespan [5-8]. LBP leads to a high number of health care consultations, utilization of secondary care interventions such as surgery is increasing and costs associated with LBP are enormous [9].

Central to clinical encounters related to LBP is the clinical examination. National and international clinical guidelines for the management of non-specific LBP are consistent in recommending diagnostic procedures to focus on the identification of red flags and exclusion of specific diseases [10]. In addition, many advocate a neurological screening or examination and some recommend a more comprehensive musculoskeletal examination [10]. These procedures serve a diagnostic purpose and are also the basis upon which clinicians can outline a management strategy. In a survey of Australian primary care clinicians (general practitioners (GPs), physiotherapists, and chiropractors), 100% routinely assessed physical impairment of their LBP patients using range of motion, neurological and orthopedic tests, muscle tests and palpation, and 99% of clinicians assessed pain. In contrast, only 7% routinely assessed psychological and social parameters [11]. Consequently, the physical examination is considered a cornerstone in the evaluation of LBP patients both in national guidelines and by individual clinicians from various backgrounds. However, evidence suggests that the validity, reliability and diagnostic accuracy of the commonly used clinical tests for LBP is low [12-16], and the ability of clinical tests to predict the prognosis of the patient is questionable [17-19]. Kent et al. systematically reviewed prognostic factors for poor recovery in non-specific LBP, including clinical tests, and concluded that uncertainty remains regarding which prognostic factors are associated with particular outcomes, the strength of those associations and the extent of confounding between prognostic factors [18]. However, they only focused on recent onset LBP and did not include patients with neurological signs. Borge et al. concluded that there is no satisfactory answer to the question of whether some physical examination tests have a prognostic value in conservative treatment of LBP [19] but focused only on chronic LBP.

The prognostic value of clinical tests in LBP has not recently been assessed systematically and no overview exists that includes both acute, recurrent, and chronic LBP, and patients with, as well as without, leg pain and/or signs of nerve root compression.

The aim of this study was to examine the extent and quality of the evidence on clinical examination findings as prognostic factors by systematically and critically reviewing the literature dealing with the association between low-tech clinical tests used in adult patients with acute, recurrent, or chronic LBP and at least one of the outcomes of pain, disability, return to work, use of health care services or medication, or global improvement.

## Methods

The Preferred Reporting Items for Systematic Reviews and Meta-Analyses (PRISMA) were used for the reporting of this systematic review [20].

### Search strategy

Using the strategy of broad search terms for systematic reviews of LBP prognosis [21], relevant articles from peer-reviewed journals were identified by computerised searches in the databases MEDLINE (from 1966), Embase (from 1974) and MANTIS (from 1888) from inception to June 26^th^, 2012. The preliminary searches were assisted by an experienced research librarian. The PubMed search used MeSH terms, subheadings, text words, combinations of search terms and Boolean operators. The Pubmed search strategy was adapted for use with the other bibliographic databases. The complete search strategy for Pubmed is included in Additional file 1.

The search was complemented by screening of the reference lists of relevant reviews and retrieved papers, bibliography screening and citation tracking of authors of relevant studies.

### Inclusion criteria

Studies had to have investigated low-tech clinical tests (tests performed without the use of equipment other than simple inexpensive devices like a handheld goniometer, a reflex hammer, a pinwheel or a tape measure), and reported the statistical association between clinical examination findings at baseline and at least one of the outcomes of pain, disability, return to work, use of health care services or medication, and global improvement. Prospective clinical studies concerning adult patients with LBP with or without leg pain and/or signs of nerve root involvement or spinal stenosis, receiving no or non-surgical treatment were included. Sample size needed to be ≥50 with a follow-up of seven days or more. Only original research manuscripts published in peer-reveiwed journals and written in English, Danish, Swedish or Norwegian were considered.

### Exclusion criteria

Studies were excluded if they did not involve clinical populations; if LBP could not be isolated from other conditions; if participants were pregnant; or if they had specific diseases such as inflammatory disease, tumor, fracture, or cauda equina. Studies were also excluded if the clinical test involved equipment that could not be expected to be generally available in primary care practice, for example equipment to measure muscle strength or aerobic capacity. Also studies in which choice of treatment was based on the results of the clinical tests were excluded because a prognostic effect could not be separated from a treatment effect.

### Screening

Screening of 300 titles was performed independently by three reviewers and another 200 titles by two reviewers in order to calibrate threshold for inclusion. The rest of the titles were screened by the first author alone (LHa). Sixty abstracts were drawn by a random sequence generator (www.random.org) and screened independently by the three authors and a research colleague. Disagreements on eligibility according to inclusion and exclusion criterias were discussed and consensus reached. The remaining abstracts and all eligible full text articles were screened independently by one of two pairs of review authors (LHa/LH; LHa/AK). Reference lists of key studies and relevant systematic reviews were screened for additional articles by the first author.

### Data extraction

The authors defined a descriptive checklist which was tested and improved before use for data extraction. Two pairs of review authors (LHa/LH; LHa/AK) extracted all relevant information. Disagreements were resolved by discussion between all three authors.

### Classification of predictors

Because of the very large number of clinical tests and the variations of tests in the included studies, we created a coding taxonomy for predictor variables to allow variables to be compared across studies despite differences in labels and measurement scales. We grouped the tests under eight headings: symptom response classification, spinal range of motion, palpation, pain provocation tests, muscle strength and endurance, neurological tests, non-organic signs, and functional tests (Additional file 2).

### Definitions of outcomes

The outcome variables were also prone to a large degree of variation in definitions and were grouped under six predefined domains: pain, disability, return to work, use of health care services or medication, global improvement and combination outcomes. Definitions and measurement scales included in the outcome domains are described in Additional file 3.

### Quality assessment

The methodological quality of the included studies was assessed independently by two reviewers using five domains of potential bias based on the work by Hayden et al. [22,23] (Table 1). The quality assessment instrument originally included six domains of potential bias, but the domain “modifying factors” was not included in this bias assessment, since our focus was on prediction rather than exploring causative associations. Futhermore, little is known about factors potentially modifying associations with clinical tests and therefore evaluation of model completeness was not possible. Quality-related questions were scored as: yes, partly, no, or not reported/unsure, which led to an overall scoring of low, moderate or high risk of bias. This approach is not based on summated scores but involves evaluating information about different designs or conduct features of the research question to ensure a more balanced judgement of each domain of potential bias. Disagreements were resolved by discussion until concensus was reached. The reviewers were not blinded.

**Table 1:** **Overview of quality domains**

**Domains of potential bias**	**Question**	**Data extraction items**
Study participation	The study sample represents the population of interest on key characteristics, sufficient to limit potential bias of the extracted results	Source population clearly defined. Study population described (inclusion and exclusion criteria). Study population represents population of interest.
Study attrition	Loss to follow-up (from target population to final study sample) is not associated with key characteristics (i.e. the study sample adequately represents the population of interest). This is sufficient to limit bias of the extracted results.	Completeness and transparency of follow-up described for each relevant point of follow-up. Completeness of follow-up adequate. Reasons for loss to follow-up are adequately described for key characteristics.There are no important differences between key characteristics and outcomes in participants who completed the study and those who did not.
Prognostic factor measurement	The prognostic factors of interest are adequately measured in study participants to sufficiently limit bias of the extracted results	Prognostic tests are defined well enough to be replicated. The performance of the prognostic tests are standardized appropriately.
Outcome measurement	The outcome of interest is adequately measured in study participants to sufficiently limit bias of the extracted results.	Outcomes are defined. Outcome measures are well established. Method, setting and time of outcome measurements are the same for all participants.
Analysis & reporting	The statistical analysis and reporting of results is transparent and appropriate in relation to the study, limiting potential for presentation of invalid results	There is sufficient presentation of data to assess the adequacy of the analysis.The statistical analysis is sufficiently described and appears appropriate in relation to the part of the study that concerns the present review. There is no selective reporting of results.

Modified from Hayden, 2006 [22].

### Data synthesis and analysis

An association was considered statistically significant if the reported p-value was <0.05 or the 95% confidence interval for a risk/odds ratio did not include 1.0. To give a broad overview of the extent of existing research in this field, both high, moderate, and low quality studies were reported. About half of the studies did not account for any modifying factors or confounders, and the studies adjusting for covariates did so with very different approaches to modelling, demonstrating a large variety in levels of complexity. This heterogeneity made direct comparisons difficult and thus, items 13 and 21 in the PRISMA protocol were not followed. We based our conclusions on the univariate or discriminant analyses when possible (a few studies did not report results from univariate analyses) and reported results of multivariable analysis when present. It has not been considered if the set of covariates in the multivariable models were adequate or meaningful. Heterogeneity of studies prevented any meaningful pooling of quantitative estimates of the associations between prognostic factors and outcome.

The strength of evidence for the reported prognostic factors is summarized using four levels of evidence: 1) c*onsistent evidence*: consistent findings in two or more studies, or at least 75% of the studies reporting similar conclusions (one of the studies should be of high quality; 2) *limited evidence*: findings in one study of high quality or two or more studies of low quality; 3) *conflicting evidence*: <75% of available studies reporting similar findings, or contradictory findings present within one study; and 4) *no evidence:* prognostic factor investigated in none or only one study of low or moderate quality [24].

## Results

### Results of the search

**Figure 1:**
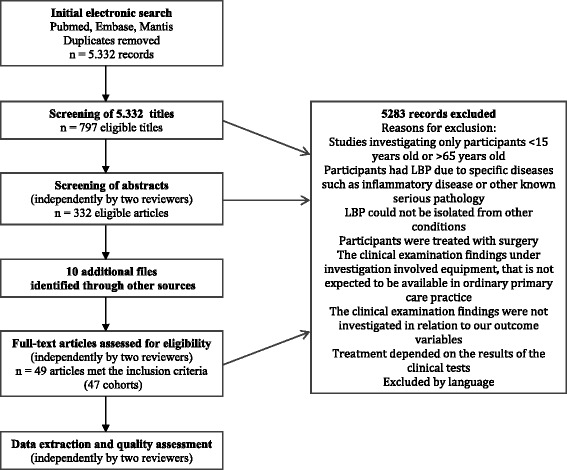
**Flow chart outlining the literature search and study selection.**

The search identified 5,332 citations. The process for selecting the eligible studies is presented in Figure 1. A total of 47 studies reported in 49 articles were included in the final review [25-73].

### Study characteristics

#### Design

All studies were prospective cohort studies, of which 13 were secondary analysis of data from randomized clinical trials. Sixteen papers reported on short-term follow-up (≤3 months), 24 on long-term follow-up (>3 months), and nine papers reported on both. Four studies were confirmatory with an objective to investigate the prognostic value of one specific test. The rest were explorative studies that looked for associations with outcome among a number of other baseline characteristics.

#### Study population

The 49 included articles represented 47 different cohorts of LBP patients and originated from Europe (*n* = 31), USA (*n* = 11), Canada (*n* = 4), Australia (*n* = 2), and one was of unknown origin.

Twenty-five studies were set in secondary care, 14 in primary care, and in three studies both primary and secondary care patients were included; in one study patients were recruited in a privately owned rehabilitation clinic, in two studies in military medical centers and in one study at factory health centres. Three studies failed to report on setting.

Twelve studies reported on patients with LBP of ≤ 3 months duration, 16 studies reported on patients with chronic LBP (>3 months duration), and 21 included mixed populations or had an unclear definition of the duration of LBP.

#### Study outcomes

Return to work and disability were the most common outcomes, used in 24 and 22 studies respectively, followed by pain (19 studies), global improvement (seven studies), use of health care services or medication (six studies), and a combination outcome (six studies).

#### Predictor variables

Straight leg raise (SLR), neurological signs, spinal range of motion, palpation, and non-organic signs were the most frequently investigated tests. The rest of the variables were investigated in six studies or less. Altogether, 26 categories of tests were identified and most of these covered several different tests or variations of the same test.

Details and characteristics of the 49 included articles can be found in Additional file 4.

### Methodological quality

The assessment of study quality (risk of bias) for all included studies is presented in Table 2. Twenty-two studies were considered to be of low quality, thirteen studies of moderate quality, and fourteen studies of high quality. The most frequently noted shortcomings related to unclear definition of source population or insufficient description of inclusion and exclusion criteria (n = 23), lack of transparency in description of each point of follow-up or inadequate follow-up (n = 27), and uncertainty of prognostic factor measurement (n = 29). In 25 studies, presentation of data to assess the analysis was only partly adequate (n = 15) or inadequate (n = 10).

**Table 2:** **Results of methodological assessment of the 49 included studies**

**Author and year of publication**	**Study participation**	**Study attrition**	**Prognostic factor measurement**	**Outcome measurement**	**Analysis/reporting of results**	**Risk of bias assessment**
Albert et al. (2012) [25]	Yes	Unsure	Yes	Yes	Yes	Low
Amundsen et al. (2000) [26]	Unsure	Partly	Unsure	No	No	High
Bendix et al. (1998) [27]	Yes	Partly	Unsure	Yes	Partly	Moderate
Bergquist-Ullman et al. (1977) [28]	Yes	Unsure	Yes	Yes	Partly	Moderate
Burton et al. (1991) [29]	Yes	Partly	No	No	Partly	High
Burton et al. (1995) [30]	Yes	Yes	No	Yes	Partly	Moderate
Campello et al. (2006) [31]	No	Yes	Partly	Yes	Yes	Moderate
Christiansen et al. (2010) [32]	Yes	Yes	Yes	Yes	Yes	Low
Coste et al. (1994) [33]	Yes	Unsure	Unsure	Unsure	Partly	High
Dwornik et al. (2007) [34]	Unsure	Unsure	Unsure	Partly	No	High
Enthoven et al. (2003) [35]	Yes	Partly	Yes	Yes	Yes	Low
Ferreira et al. (2009) [36]	Partly	Unsure	Yes	Yes	Yes	Moderate
Flynn et al. (2002) [37]	Yes	Yes	Yes	Partly	Yes	Low
Fritz et al. (2004) [40]	Yes	Yes	Yes	Partly	Yes	Low
Fritz et al. (2007) [38]	Unsure	Yes	Yes	Yes	Yes	Low
Fritz et al. (2005) [39]	Yes	Yes	Yes	Yes	Yes	Low
Gaines et al. (1999) [41]	Yes	Unsure	Yes	Partly	Yes	Moderate
Ghahreman et al. (2011) [42]	Yes	Unsure	Unsure	Yes	Partly	High
Grotle et al. (2005) [44]	Unsure	Yes	Unsure	Yes	Partly	Moderate
Grotle et al. (2007) [43]	Unsure	Yes	Unsure	Yes	Partly	Moderate
Gurcay et al. (2009) [45]	Partly	Yes	Unsure	Yes	Partly	Moderate
Hicks et al. (2005) [46]	Unsure	Yes	Yes	Yes	Yes	Low
Hildebrandt et al. (1997) [47]	Partly	Yes	Unsure	Partly	Partly	High
Hurri et al. (1989) [48]	No	Partly	Unsure	Yes	Yes	High
Indahl et al. (1998) [49]	Unsure	Yes	Partly	Unsure	Partly	High
Infante-Rivard et al. (1996) [50]	Yes	Partly	No	Unsure	Yes	High
Jamison et al. (1991) [51]	Unsure	No	Unsure	Yes	No	High
Karas et al. (1997) [52]	Yes	Partly	Yes	Yes	Yes	Low
Kool et al. (2002) [53]	Unsure	Yes	Yes	Partly	Yes	Moderate
Leboeuf-Yde et al. (2004) [54]	Yes	Partly	Unsure	Yes	Yes	Low
Long et al. (1995) [55]	Yes	Unsure	Yes	Partly	Partly	High
Lonnberg (2010) [56]	Unsure	Unsure	Unsure	Yes	Yes	High*
Luoto et al. (1998) [57]	Unsure	Yes	No	No	No	High
McIntosh et al. (2000) [58]	Unsure	Yes	Unsure	Yes	Yes	Moderate
Milhous et al. (1989) [60]	No	Partly	No	Partly	No	High
Michaelson et al. (2004) [59]	Yes	Yes	Partly	Yes	Yes	Low
Pedersen (1980) [61]	Yes	Yes	Yes	Unsure	No	High
Polatin et al. (1989) [62]	Unsure	Unsure	Unsure	Yes	Partly	High
Roland (1983) [63]	Unsure	Yes	Yes	Unsure	No	High
Sandström et al. (1986) [64]	Yes	Yes	Partly	Yes	No	High
Schiøtt-Christensen et a (1999) [65]	Yes	Yes	Unsure	Yes	Yes	Low** Medium***
Seferlis et al. (2000) [66]	Unsure	Partly	Partly	Partly	Yes	High
Skytte et al. (2005) [67]	Yes	Unsure	Yes	Yes	Partly	Moderate
Sweetman et al. (1996) [68]	Unsure	Unsure	Unsure	Unsure	No	High
Valls et al. (2001) [29]	Yes	Unsure	Unsure	Partly	Yes	Moderate
Van den Hoogen et al. (1997) [70]	Yes	Partly	Yes	Yes	Yes	Low
Vendrig et al. (1999) [71]	Yes	Partly	Unsure	Yes	No	High
Vroomen et al. (2002) [72]	Unsure	Yes	Yes	Yes	Yes	Low
Werneke et al. (1993) [73]	Yes	Unsure	Yes	Partly	Partly	High

*for our purpose, **for 1 + 6 months follow-up, ***for 1 year follow-up.

### Associations between prognostic factors and outcomes

An overview of the associations between prognostic factors and outcomes is presented in Table 3.

**Table 3:** **Associations between prognostic factors and outcome**

**Prognostic indicator at baseline**	**Association with poor outcome**	**Pain**	**Disability**	**Return to work**	**UHC** ^**1**^	**GI** ^**2**^	**Combination**
Centralization	Positive	**[25]** ^**a**^[55]^a^ [67]^a^	**[25]** ^**a**^[38]^a^[67]^a^				
short-term	None	[67]	[55][37]				
Centralization	Positive	**[25]** ^**a**^	**[25]** ^**a**^[67]^a^	[55]^a^	[67]^a^		
long-term	None	[67]**[32−/−]**	[67][55]**[32−/−]**	[67][55]**[32−/−]**	[67]		
Peripheralization	Positive	**[25]** ^**a**^	**[25]** ^**a**^				
short-term	None		[38]				
Peripheralization	Positive	**[25]** ^**a**^	**[25]** ^**a**^				
long-term	None	**[32−/−]**	**[32−/−]**	**[32−/−]**			
Palpation tone, pain, symmetry	Positive						
short-term	none	[54]	[37][54]			[72][68]	**[45−/−]**
Palpation tone, pain, symmetry	Positive		**[48]** ^**b**^				
long-term	none	[54]	[54]	[64]	[69]		
Palpation mobility	Positive		**[46+/+]** ^**a**^ **[37+/+]** ^**a**^				
short-term	none	**[36?/-]**	[39][38]**[36?/-]**			**[36?/-]**	
ROM^3^ spine	Positive						**[29]** ^**a**^
short-term	None	**[47?/-]**	[37][46][38]	[33] **[47?/-]**		[68]**[47?/-]**	**[29]**[33]
ROM^3^ spine	Positive	[35][28]	**[48]** ^**b**^	**[50+/+][62+/?]** ^**a**^			**[29]**
long-term	None	**[63?/-]**	[35]**[30]** ^**b**^[66]	[28][31][50] [62][66][64]	**[69+/−]**		[65]**[29]**
FFD^4^	Positive					**[72−/+]** ^**a**^	**[45+/−]**
short-term	None	**[47?/-]**	**[44−/−]**	**[47?/-]**		**[47?/+]**[68][72]	
FFD^4^	Positive			**[49+/−]**			
long-term	None	[35]	[35]**[43−/−]**				
Schober’s test	Positive	**[70+/−]** ^**b**^					
long-term	None	[28]		[28]	[69]		
Aberrant spinal movement	Positive	**[46+/+]** ^**a**^	**[33+/−]**				
short-term	None						[33]
ROM^3^ of the hip	Positive		**[37 − +/+]**				
short-term	None					[68]	**[29]**
ROM^3^ of the hip	Positive			[62]^b^			**[29]**
long-term	None	**[63−/−]**		[31]			**[29]**
SI^5^ motion symmetry tests	Positive						
short-term	None		[37]				
Pain on spinal movement	Positive					[68]^a^	
short-term	None	[54]	[54]			[68]	
Pain on spinal movement	Positive						
long-term	None	[54][56]	[54][56]		[56]		
SI^5^ provocation tests	Positive		**[40+/+]**				
short-term	None		[37][46][40]				
SI^5^ provocation tests	Positive						
long-term	None	**[63?/-]**					
Prone instability test	Positive		**[46+/+]** ^**a**^				
short-term	None						
Percussion	Positive						
short-term	None						[65]
Percussion	Positive						
long-term	None						[65]
Muscle endurance	Positive						
short-term	None	[59]	[46]				
Muscle endurance	Positive	[35]**[27]**		**[27]**			
long-term	None	[35][59]**[27]**	[35][27][57]	**[27]**[31]		[27]	
Muscle strength	Positive						
short-term	None		[46]				
Muscle strength	Positive	[28]	**[48]** ^**b**^	[28]			
long-term	None			[31]			
Neurological signs	Positive	[44]	**[44+/+]**	[63]		**[72−/+]**[68]	**[29]**
short-term	None	[65][51][42]		[63]		[72]	**[29]**
Neurological signs	Positive	**[43+/−][63?/+]**	**[43+/−][30]** ^**b**^	**[58+/−]**			**[29]**
long-term	None	[28]**[63?/-]**	**[43−/−]**	[58][28][60][50][64]	[69]		[65]**[29]**
SLR^6^	Positive		**[46−/+]**[63]	**[65+/−]**[63]		**[72+/+]**[68]	**[29]**
short-term	None	[51]		[33]		[72][26]	**[45−/−][29]**[33]
SLR^6^	Positive		**[30]** ^**b**^[61]		**[69+/−]**		**[29]**
long-term	None	[28][35][56]**[70−/−][63?/-]**	[56][66][35]	[58][28][60][66][64]	[56]	[26]	[65]
Cross SLR^6^	Positive	[34]					
short-term	None		[38]				
Cross SLR^6^	Positive						
long-term	None	**[63?/-]**					
Femoral stretch	Positive					**[72+/+]**[68]	
short-term	None					[72]	
Femoral stretch	Positive						
long-term	None			[58]			
Naffziger sign	Positive						
short-term	None					[72]	
Non-organic signs	Positive			[73]			**[29]**
short-term	None		[37]				**[29]**
Non-organic signs	Positive			**[52+/+][41+/?]**	**[41+/?]**		
long-term	None		**[30]** ^**b**^	**[53+/−][58+/−]**[71]	**[41+/?]**		**[29]**
Functional tests	Positive						
short-term	None					[68]	
Functional tests	Positive						**[29]**[61]
long-term	None	[27]	**[30]** ^**b**^[27]	[31][27][62]		[27]	**[29]**
Leg length discrepancy	Positive						**[29]**
short-term	None						**[29]**
Leg length discrepancy	Positive						
long-term	None			[64]			

Bold indicates multivariable analysis, +/+ = result of univariate analysis/result of multivariable analysis, ^a^predictor of recovery, ^b^direction of association unsure, ^1^UHC = use of health care services or medication, ^2^GI = general. improvement, ^3^ROM = range of motion, ^4^FFD = fingertip to floor distance, ^5^SI = sacroiliac, ^6^SLR = straight leg raise.

### Detailed findings

#### Symptom response classification – short-term outcome

Five studies reported on symptom response classification: three of high quality [25,37,38], one of moderate quality [67], and one of low quality [55].

##### High quality studies

One study found centralization to be a prognostic factor for less pain (only multivariable analysis reported) [25] and two for less disability [25,38]. One study found no association with disability [37]. In this study, centralization was tested with single movement testing which is not the traditional way of using the test. Peripheralization was found to be a prognostic factor for less pain and disability in one study [25], whereas another study tested peripheralization with extension and found no association with disability [38]. All three studies included mixed populations, one in secondary care [25], one in primary care [38], and one in military medical centers [37].

##### Low and moderate quality studies

One study of moderate quality including acute secondary care patients found centralization to be associated with less disability at one, two and three months follow-up and for less pain at two and three months follow-up but not at one month follow-up [67]. One low quality study found an association with better outcome of pain but not disability in a chronic primary care population [55].

#### Symptom response classification – long-term outcome

Two high quality studies [25,32], one moderate quality study [67], and one low quality study [55] reported on symptom response classification in relation to long-term prognosis.

##### High quality studies

One study found centralization and peripheralization to be associated with better outcome of pain and disability (only multivariable analysis reported) [25], and another study found no association with pain, disability, or return to work in either univariate or multivariable analyses [32]. Both studies were on mixed populations in secondary care settings with a one year follow-up.

##### Low and moderate quality studies

One moderate quality study included acute patients in a secondary care setting and found no association between centralization and pain or return to work at six months or one year but did find it to be a prognostic factor for less disability at one year [67]. The same study showed that patients who did not centralize were six times as likely to have surgery but found no association with use of medication. One low quality study included chronic patients in a primary care setting and found no association between centralization and disability [55]. The same study found centralization to be a prognostic factor for a better outcome of return to work at nine months but although the trend still existed at two years follow-up, it was not significant.

#### Summary of symptom response classification

Two confirmatory studies were explicitly designed to test the prognostic capacity of centralization, one tested centralization as a prognostic factor (in a univariate analysis) [67] and the other explored it as an independent prognostic factor [55]. There is consistent evidence for centralization being a prognostic factor for less short-term pain and conflicting evidence for an association with disability. Re-scaling disability scores to 0–100 scales, centralizers improved on average 7 points (range −2 to 14) more than non-centralizers across short-term time points (55,67,38), and 28 points more than patients neither centralizing nor peripheralizing (25). Using a dichotomized outcome, the proportion having a successful outcome was no different in centralizers than in the total cohort; whereas the prevalence of success among those with peripheralizing symptoms was 17 percentage points lower than in the total cohort (28% versus 45% success) (37). Re-scaling pain scales to 0–100, centralizers reported 9 points (range 5–14) more improvement than non-centralizers (55,67) and 23 points more than patients neither centralizing nor peripheralizing (25).

The evidence for an association between centralization and long-term outcome is conflicting, as is the evidence for peripheralization in association with both short-term and long-term outcome. Setting, duration of LBP at baseline, sample size, or method of outcome measurement did not explain the variation in results.

### Palpation

#### Palpation for tone, pain or asymmetry – short-term outcome

Three high quality studies [37,54,72], and one of low quality [68] reported on palpation for pain. One study of moderate quality reported on palpation for muscle spasm (both univariate and multivariable analyses) [45], and one high quality study investigated palpation for asymmetry [37]. None of the studies found palpation to be associated with outcome. Studies included both acute, subacute and mixed populations from both primary and secondary care settings.

#### Palpation for tone, pain or asymmetry – long-term outcome

Four studies investigated palpation for tone or pain, one high quality [54], one moderate quality [69], and two low quality studies [48,64].

##### High quality studies

The high quality study found no association between pain on palpation and the outcomes of pain and disability at one year in a subacute primary care population [54].

##### Low and moderate quality studies

The moderate quality study found no association between radicular pain on palpation using finger pressure on the paraspinal area and the one year outcome of use of health care services or medication [69]. One low quality study found no association between increased tonus on palpation of paraspinal muscles and return to work at one year follow-up [64]. Another low quality study found the number of painful spots on palpation in the lumbar area as well as the number of painful spots in the shoulder-neck area to be associated with disability after one year [48]. The low and moderate quality studies were all set in secondary care, two included chronic patients [48,64] and one a mixed population [69].

#### Palpation for mobility – short-term outcome

Four high quality [37-39,46] and one moderate quality study [36] reported on palpation for spinal mobility.

##### High quality studies

One primary care study found the absence of hypermobility on the springing test to be a prognostic factor in patients who received a stabilization program [46], and another study found segmental hypomobility on palpation to be prognostic of good outcome in patients who received a manipulation treatment program at military medical centers [37]. Both were acute/subacute patient populations. The results were maintained in multivariable analyses. Two studies found no association between segmental hyper/hypomobility and disability [38,39]. Both of these were set in outpatient practices, although one study also included patients from two academic medical centers [39].

##### Low and moderate quality studies

One moderate quality study on chronic non-specific LBP patients in secondary care reported on spinal stiffness and found no association with pain, disability or global improvement (only multivariable analysis) [36]. One low quality study dealt with palpation but failed to report results [34].

Setting, duration of symptoms at baseline, sample size, or method of outcome measurement did not explain the heterogeneous results.

#### Palpation for mobility – long-term outcome

No studies investigated the association between palpation for mobility and long-term outcome.

#### Summary of palpation

High quality studies consistently show no evidence for an association between palpation for pain, tone or symmetry and short-term or long-term outcome. There is conflicting evidence for an association between palpation for mobility and short-term outcome. Palpation for mobility as a prognostic factor for long-term outcome is not investigated.

### Range of motion tests (ROM)

#### Spinal ROM – short-term outcome

Ten studies investigated spinal ROM: three of high quality [37,38,46], one of moderate quality [44], and six of low quality [29,33,34,47,63,68].

##### High quality studies

Three high quality studies investigated spinal ROM and found no association with disability, one in a subacute population [46], and two in a mixed population [37,38]. All three studies were conducted in primary care type settings.

##### Low and moderate quality studies

Two studies reported on flexion/extension [29,68] and two on spinal ROM without specification [33,47] and found no association with outcome. One study reported on extension and found no association with overall improvement at one month or at three months, but did find extension to be associated with the outcome of being symptom-free at one month [29]. One study showed that decreased flexion was associated with a better chance of overall improvement [29], and one study stated that “flexion was most strongly related to outcome” but the direction and statistical significance of the association was unclear [63]. One low and one moderate quality study dealt with spinal ROM but failed to report any results [34,44].

Two studies included acute primary care patients [33,44], one chronic secondary care patients [47], one study included a mixed population in a mixed setting [29], one included a mixed population in an unknown setting [34], and two studies failed to report on duration of symptoms [63,68].

#### Spinal ROM – long-term outcome

Fourteen studies dealt with spinal ROM in relation to long-term outcome, two were of high quality [35,65], five of moderate quality [28,30,31,43,69], and seven were low quality studies [29,48,50,62-64,66].

##### High quality studies

Two studies reported on spinal ROM without specification and thoracolumbar rotation respectively and found no association with pain and disability [35,65].

##### Low and moderate quality studies

Five studies investigated flexion. Three low quality studies found spinal flexion to be associated with outcome [48,50,62], however, results of these were not in the same direction. One study found reduced flexion to be more dominant in the “success group” relative to the “failure group” [62], and another study found that good flexion at baseline was associated with good outcome of return to work [50], which was retained in the final multivariable model. One study of moderate quality [31] and one of low quality [29] did not find any association between flexion and outcome. Three studies reported on flexion/extension [29,62,66], four on spinal ROM without specification [30,50,64,69] and five on other directions of spinal movement [28,29,31,48,63]. Only two of these found an association with outcome; one with disability [48], and one study found that none of the patients who had normal ROM at admission required radical treatment [69]. This result was not retained in a multivariable analysis. One study of moderate quality failed to report on results [43].

Of all studies reporting on spinal ROM, six studies were set in primary care [29,30,35,43,63,65] and seven studies in secondary care [31,48,50,62,64,66,69]. One study was set in factory health centers [28]. Five studies included acute patients [30,43,63,65,66], one study included acute/subacute patients [28], four studies were on chronic patients [31,48,62,64], and three studies included mixed populations [29,35,69]. The mixed results concerning flexion were not explained by setting, duration of symptoms at baseline, sample size, or method of outcome measurement.

#### Fingertip to Floor Distance (FFD) – short-term outcome

Five studies reported on FFD, one of high quality [72], two of moderate quality [44,45], and two of low quality [47,68].

##### High quality studies

The high quality study found FFD of >24 cm to be a predictor of better outcome of global improvement at two weeks follow-up in a multivariable analysis but not in a univariate analysis and found no association at 12 weeks follow-up [72]. This study included patients from primary care who presented for the first time with an episode of sciatica. Duration of symptoms was unclear.

##### Low and moderate quality studies

One study of moderate quality including acute patients from secondary care found a positive association between FFD and non-recovery (a combination outcome of pain and disability) however this was not sustained in a multiple logistic regression analysis [45]. Another moderate quality study on acute primary care patients found no association with disability and failed to report on other outcomes in the study [44]. One low quality study including chronic secondary care patients found an association between FFD and poor outcome of global improvement, but failed to report on associations with the outcomes of pain and return to work [47]. One low quality study found a limited FFD to be associated with poor outcome of global improvement [68]. This study failed to describe setting and duration of symptoms.

The studies reporting an association between FFD and outcome had follow-up of two weeks (*n* = 2), eight weeks and “short-term”, whereas the two studies looking at a follow-up of three months found no association between FFD and outcome. Setting, duration of symptoms at baseline, sample size, and method of outcome measurement did not explain the variation in results.

#### Fingertip to Floor Distance (FFD) – long-term outcome

Three studies reported on the association between FFD and long-term outcome, one of high quality [35], one of moderate quality [43], and one of low quality [49].

##### High quality studies

No association was found between FFD and pain and disability in a mixed primary care population [35].

##### Low and moderate quality studies

The moderate quality study included an acute primary care population and found no association with disability but failed to report on other outcomes in the study [43]. The low quality study found greater FFD to be predictive of poor outcome of return to work [49]. It included subacute patients in a secondary care setting and had a follow-up of five years [49].

#### Schober’s test – short-term outcome

One low quality study dealt with Schober’s test but failed to report any results regarding its prognostic value [34].

#### Schober’s test – long-term outcome

Three studies reported on the modified Schober’s test, one of high quality [70], and two of moderate quality [28,69].

##### High quality studies

The high quality study included a mixed primary care population and found an association between Schober’s test and time to recovery from pain [70], however, the association did not hold in the multivariable analysis.

##### Moderate quality studies

Two moderate quality studies found no association with outcome [28,69].

Both studies included mixed populations, one in secondary care, and one in factory medical centers. Sample size and method of outcome measurement did not explain the heterogeneity in results.

### Aberrant spinal movement – short-term outcome

One high quality and one low quality study reported on aberrant spinal movement [46,33]. The high quality study found that the presence of aberrant spinal movement in a subacute population was prognostic of less disability, and absence of aberrant spinal movement was prognostic of more disability in patients who received a stabilization program [46]. The association was maintained in a multivariable analysis. The low quality study found an association with poor outcome of return to work and no association with a combination outcome of pain and disability in an acute population [33]. The positive association with return to work disappeared in the multivariable analysis. Both studies were set in primary care.

### Aberrant spinal movement – long-term outcome

No studies investigated the association between aberrant spinal movement and long-term outcome.

#### ROM of the hip – short-term outcome

Four studies investigated ROM of the hip, one of high quality [37], and three of low quality [29,63,68].

##### High quality studies

One high quality study investigating hip rotation in acute or subacute patients found that left but not right internal hip rotation was predictive of a better outcome of disability. In the multivariable analysis having >35 degrees of internal rotation in at least one hip was predictive of better outcome of disability [37]. The study was set in military medical centers.

##### Low quality studies

One low quality study included a mixed primary care population and found no association between ROM of the hip and a combination outcome of pain and disability [29]. Another low quality study found no association between ROM of the hip and global improvement [68]. This study failed to report on setting and duration of symptoms. One low quality study dealt with ROM of the hip but did not report results [63].

#### ROM of the hip – long-term outcome

Four studies reported on ROM of the hip. One was of moderate quality [31], and three were of low quality [29,62,63].

##### Low and moderate quality studies

The study of moderate quality found no association between ROM of the hip and return to work at two years follow-up in a chronic secondary care population [31].

One low quality study included a mixed population in a primary care setting and found that passive flexion/adduction of the hip was associated with the outcome of being symptom-free but not overall improvement [29]. A low quality study set in secondary care included chronic patients and found an association between hip flexion and return to work [62], whereas another low quality study set in primary care included acute patients and found no association between hip rotation and pain [63].

### Sacroiliac (SI) motion symmetry tests – short-term outcome

One high quality study reported in two papers found no association between SI motion symmetry tests and short-term outcome [37,40].

### SI motion symmetry tests – long-term outcome

No studies investigated SI motion symmetry tests in relation to long-term outcome.

#### Summary of ROM

High quality studies consistently show no evidence for an association between spinal ROM without specification and short-term or long-term outcome. For FFD, there is conflicting evidence in relation to the test as a prognostic factor for short-term outcome and limited evidence in relation to long-term outcome. For Schober’s test, which is also a flexion measure, there is conflicting evidence for the test being a prognostic factor for long-term outcome. We did not find any studies investigating Schober’s test in relation to short-term outcome.

There is limited evidence for aberrant movement on spinal ROM being a prognostic factor for short-term outcome, and no studies on the association with long-term outcome. For SI motion tests evidence is limited and shows no evidence of an association with short-term outcome. No studies investigated the long-term prognostic value of SI motion tests. There is conflicting evidence for the association between ROM of the hip and short- and long-term outcome. This heterogeneity was not explained by setting, duration of symptoms, sample size, or method of outcome measurement.

### Pain on spinal movement – short-term outcome

One high quality and one low quality study investigated pain on spinal movement [54,68]. The high quality study was set in primary care and included subacute patients. This study found no association with pain and disability [54]. Duration of pain and setting in the low quality study was unclear. They found pain on supine extension to be an indicator of poor outcome of global improvement and found no association between pain on standing extension and outcome. The same study found pain in the end-range of flexion to be associated with poor outcome of global improvement but found no association between pain on flexion, on lateral flexion and on rotation and global improvement [68].

### Pain on spinal movement – long-term outcome

One high quality and one low quality study reported on pain on spinal movement and found no association with outcome after one year and 22 years respectively [54,56]. Both studies were set in primary care. The high quality study included subacute patients [54], and the low quality study included a mixed population [56].

#### Summary of pain on spinal movement

For pain on spinal movement there is conflicting evidence for an association with short-term outcome and evidence consistently shows no evidence for an association with long-term outcome.

### Pain provocation tests

#### SI pain provocation tests – short-term outcome

Two high quality studies reported on SI provocation tests in three papers [37,40,46]. All reported on the outcome disability. The study reported in two papers found six out of seven SI pain provocation tests not to be associated with outcome, but in one paper [40] a positive Gainslen’s test was found to be associated with poor outcome of disability (maintained in the multivariable analysis), whereas the other paper reported no association with disability [37]. The study was set in military medical centers and included a mixed population. Another study found no association between posterior shear test and disability [46]. This study included subacute patients in primary care and an outpatient clinic at an airforce base. One low quality study stated that strain to SI ligaments was of no prognostic value, but failed to describe which outcomes they were referring to [63].

#### SI pain provocation tests – long-term outcome

One low quality study reported on pain on straining the anterior and posterior SI ligaments and found no association with the probability of recurrence of pain within one year in an acute primary care population [63].

#### Prone instability test – short-term outcome

One high quality study found that a positive prone instability test was prognostic of good outcome of disability in patients who received a stabilization program [46]. The association was retained in a multivariable analysis. This study included subacute patients in three outpatient physiotherapy clinics and an outpatient clinic at an airforce base.

#### Prone instability test – long-term outcome

No studies investigated the prone instability test as a prognostic factor for long-term outcome.

#### Percussion test – short- and long-term outcome

One high quality study including acute primary care patients reported on both short- and long-term outcome and found no association between percussion test and a combination outcome of pain and disability [65].

#### Summary of pain provocation tests

For SI pain provocation tests there is consistently no evidence for an association with short-term outcome except for Gainslen’s test for which there is conflicting evidence. There is no evidence for an association with long-term outcome. For prone instability test evidence is limited and shows no evidence of an association with short-term outcome. We did not find any studies investigating the long-term prognostic value of the test. Evidence is limited and shows no evidence of an association between the percussion test and short-term or long-term outcome.

### Muscle strength and endurance

#### Muscle strength and endurance – short-term outcome

Two high quality studies reported on muscle strength tests [46,59], and one on muscle endurance tests [46], and none of them found these tests to be predictive of pain and disability. One study included subacute patients from primary care [46], the other included chronic patients in secondary care [59].

#### Muscle endurance – long-term outcome

Five studies reported on muscle endurance, two high quality [35,59], two moderate quality [27,31], and one low quality study [57].

##### High quality studies

One study on primary care patients reported on high endurance of back flexors and found no association with disability but did find it to be associated with a decrease in pain after one year [35]. The same study found no association between endurance of the back extensors and outcome [35]. Another study on chronic patients in secondary care looked at endurance as an index based on sit-ups, back extensions, and hip extensions and found no association with pain [59].

##### Low and moderate quality studies

One moderate quality study on chronic secondary care patients investigated endurance of the back flexors and found no association with outcome [27]. Three studies reported on endurance of the back extensors. One study of moderate quality found an association between low endurance of the back extensors and poor outcome of return to work (disability pension), and between high endurance of back extensors and poor outcome of back pain but no association with leg pain, disability or global improvement [27]. One low quality and one moderate quality study found no association with outcome [31,57]. One study tested repetitive squatting and found it to be of no prognostic value [57].

Except for one high quality study which included a mixed primary care population [35], all studies on muscle endurance tests were set in secondary care and included chronic patients. The heterogeneous results were not explained by setting, duration of symptoms, sample size, or method of outcome measurement.

#### Muscle strength – long-term outcome

Two studies of moderate quality found no association between muscle strenght tests and outcome [28,31], one included acute patients in factory health centers [28], the other chronic secondary care patients [31]. One study of low quality found an association between muscle strength and disability in chronic secondary care patients [48]. Setting, duration of symptoms at baseline, sample size, or method of outcome measurement did not explain the results.

#### Summary of muscle strength and endurance

There is consistently no evidence of an association between muscle endurance and short-term outcome. Regarding long-term outcome, we consistently found no evidence for an association with disability and conflicting evidence in relation to pain and return to work. For muscle strength evidence is limited and shows no evidence of an association with short-term outcome. There is conflicting evidence in relation to long-term outcome.

### Neurological tests

#### Neurological signs – short-term outcome

Nine studies dealt with neurological signs, two of high quality [65,72], two of moderate quality [44,45], and five of low quality [29,42,51,63,68].

##### High quality studies

Both high quality studies included primary care patients and showed neurological signs to have no univariate prognostic value [65,72]. However in one of these, including a mixed primary care population, hypaesthesia was found to be a prognostic indicator in a multivariable analysis at two weeks but not at three months follow-up [72].

##### Low and moderate quality studies

One moderate quality study on acute primary care patients found neurological signs to be predictive of poor outcome of pain and disability [44]. This was retained in the multivariable analysis. A positive sign was defined as two or more positive tests. One low quality study found neurological signs to be predictive of poor outcome of global improvement [68], whereas two low quality studies found no association between neurological signs and outcome [42,51] and two reported mixed results [29,63]. One of them found an association between abnormal neurological signs and poor outcome of return to work but failed to report on other outcomes in the study, and furthermore, it included other definitions of neurological signs on which it did not report any results [63]. The other study found no association between sensory changes or motor changes in the leg and outcome. However, it did find “nerve root tension tests” to be associated with the outome being symptom-free at three months but not with global improvement at three months or at one month [29]. One study of moderate quality failed to report results on neurological signs [45].

Three studies reporting on neurological signs included acute patients [44,63,65], one included chronic patients [51], and three included mixed populations [29,42,72]. In one study, duration of symptoms was unclear [68]. Four studies were set in primary care [44,63,65,72], two in secondary care [42,51], and one included patients from both primary and secondary care [29]. Setting, duration of symptoms, sample size, or method of outcome measurement did not explain the variation in results.

#### Neurological signs – long-term outcome

Twelve studies investigated neurological signs, one of high quality [65], five of moderate quality [28,30,43,58,69], and six of low quality [29,50,60,61,63,64].

##### High quality studies

One high quality study on acute primary care patients found no association between neurological signs and long-term outcome [65].

##### Low and moderate quality studies

Five studies found neurological tests not to be associated with outcome. [28,50,60,64,69], four reported mixed results [29,43,58,63], and one study of moderate quality found an association between “root tension” and disability [30]. One low quality study found unilateral abnormality of reflexes to be predictive of recurrences of pain, and both strength and abnormal neurological signs to have no prognostic value [63]. One study of moderate quality found that patients with two or more positive neurological signs showed significantly less improvement in pain and disability after one year, but the association was not retained in a multivariable analysis. They found no association with “recovery”, defined as ≤ 4 on Roland Morris Disability Questionnaire [43]. One low quality study found no predictive value of sensory changes and motor changes in the leg [29]. They did find nerve root tension tests in combination with other clinical tests to be associated with being symptom-free but not with overall improvement. One study of moderate quality investigated L4, L5, S1 neurological signs and found S1 to be associated with poor outcome of return to work [58]. The association was not sustained in the multivariable analysis. One study failed to report any results [61].

Five studies included acute patients [30,43,50,63,65], two included acute/subacute patients [28,58], three included a mixed population [29,60,69], and one included chronic patients [64]. Five studies were set in primary care [29,30,43,63,65], five in secondary care [50,58,60,64,69], and one study was set in factory health centers [28].

#### Straight leg Raise (SLR) – short-term outcome

SLR as a prognostic indicator was investigated in 12 studies, five of high quality [37,38,46,65,72], one of moderate quality [45], and six of low quality [26,29,33,51,63,68].

##### High quality studies

One study found SLR to be a prognostic indicator of poor outcome of return to work although the association was not retained in a multivariable analysis [65]. This study did not define what constituted a positive test. Another study defined a positive test as ”typical dermatomal pain upon raising the leg” and showed a positive association with poor outcome of global improvement at three months, but no association at two or four weeks [72]. The association at three months was retained in a multivariable analysis. One study showed that in a multivariable analysis average SLR >91 degrees was prognostic of less disability in patients who received a stabilization program [46]. There was no association with outcome in the univariate analysis. Two studies found no association between SLR and disability [37,38]. These studies, however, were not useful for evaluation of SLR as one only included patients with a SLR of ≥45 degrees [37] and the other, only patients with a positive SLR <45 degrees [38].

##### Low and moderate quality studies

One moderate quality [45] and one low quality study [33] found no association between SLR and a combination outcome of pain and disability. One of these offered no definition of a positive test [45], whereas the other defined a positive test as “<75 degrees” [33]. This study found no association with return to work either. One low quality study that did not define a positive test, reported no association with global improvement [26], and one defined a test to be positive if radicular pain was reproduced at less than 70 degrees and found no association with pain [51]. One low quality study investigated two variations of a positive test: limited SLR with no clear definition of “limited”, and painful end point to SLR. Both were associated with poor outcome of global improvement [68]. One low quality study defined a positive test as “<60 degrees” and showed an association with disability but failed to report on other outcomes in the study [63]. This study also reported on SLR plus dorsiflexion of the foot and found an association with disability and return to work but failed to report on pain. One low quality study found an association with the outcome being symptom-free but not with global improvement at three months follow-up and no association with either outcome at one month follow-up [29]. In this study, a positive test was defined as leg pain at <50 degrees.

Four studies included acute patients [33,45,63,65], one study subacute patients [46], two studied chronic patients [26,51], fours studies included mixed populations [29,37,38,72] and in one study, duration of symptoms was unclear [68]. Seven studies were set in primary care [29,33,38,46,63,65,72], three studies in secondary care [26,45,51], and one was at military medical centers [37]. In one study, the setting was unclear [68]. All studies showing a positive association with poor outcome included acute patients or mixed populations. Most of the studies finding an association between SLR and outcome included larger cohorts and had follow-ups of two or three months, whereas five out of eight studies finding no association had follow-ups of one month or less. Setting and method of outcome measurement did not explain the heterogeneity. Definitions of a positive test varied to an extent that could affect the results.

#### SLR – long-term outcome

Fifteen studies reported on SLR, three of high quality [35,65,70], four of moderate quality [28,30,58,69], and eight of low quality [26,29,56,60,61,63,64,66].

##### High quality studies

The three high quality studies found no association between SLR and outcome [35,65,70]. In one study, a positive test was pain at <60 degrees [35], in one study SLR was considered positive if it evoked pain in the leg below the knee [70], and in one study there was no definition of a positive test [65].

##### Low and moderate quality studies

Eight studies found no association between SRL and outcome [26,28,56,58,60,63,64,66]. Two studies found SLR to be predictive of poor outcome of disability [30,61], one study found SLR to be predictive of poor outcome of use of health care services or medication, but the result was not retained in the final model [69], and one found SLR to be predictive of a combination outcome of pain and disability [29]. Six studies failed to define what constituted a positive test [26,28,30,56,58,60]. One defined it as “<50 degrees” [29], one defined a positive test as “<60 degrees” [63], in one study SLR was considered positive when pain occured in the back and leg and the range of motion was limited [64], and in one study SLR was considered positive if the patient experienced pain or resistance at ≤60 degrees. One study referred to a definition by Forst without detailing the reference [66].

Four studies included acute patients [30,63,65,66], two included acute/subacute patients [28,58], seven included a mixed population [29,35,56,60,61,69,70], and two studies included chronic patients [26,64]. Eight studies were set in primary care [29,30,35,56,61,63,65,70], six in secondary care [26,58,60,64,66,69], and one study was set in factory health centers [28]. Setting, duration of symptoms, sample size, or method of outcome measurement did not explain the variation in results.

### Cross SLR – short-term outcome

Three studies investigated crossed SLR [34,38,63].

One high quality study included a mixed primary care population and reported crossed SLR to be predictive of less change in disability [38]. However, the study had a focus on effect moderation and did not report whether an observed association in one treatment group between crossed SLR and disability was statistically significant. One low quality study on a mixed population found an association between higher crossed SLR score (degrees) and better outcome of pain [34]. This study did not report on setting. One low quality study failed to report results of the test [63].

### Femoral stretch – short-term outcome

Two studies reported on femoral stretch test [68,72]. One high quality study included acute primary care patients and found femoral stretch test to be a predictor of poor outcome of global improvement at three months but not at two weeks [72]. The association at three months was retained in a multivariable analysis. A low quality study found pain on femoral stretch test to be associated with poor outcome of global improvement [68]. This study failed to report on duration of symptoms and setting.

### Crossed SLR and femoral stretch test – long-term outcome

One low quality study on acute primary care patients found no association between crossed SLR and pain [63], and one study of moderate quality including acute/subacute patients in secondary care found no association between femoral stretch test and return to work [58].

### Naffziger sign – short-term outcome

One high quality study including a mixed primary care population found no association between Naffziger sign and global improvement [72].

### Naffziger sign – long-term outcome

No studies investigated long-term prognostic value of Naffziger sign.

#### Summary of neurological tests

Most of the studies reporting on neurological tests in association with long-term outcome were of low or moderate quality. Evidence for an association with short-term outcome is conflicting but high quality studies are rather consistent in showing no evidence of an association with outcome. For long-term outcome evidence is consistent and shows no evidence for neurological signs being prognostic factors.

The evidence for SLR, crossed SLR, and femoral stretch test as prognostic factors for short-term outcome is conflicting. There is a tendency towards an association with poor outcome at two to three months compared with just a few weeks follow-up. High quality studies consistently show no evidence of an association between SLR and long-term outcome, but overall evidence is conflicting. There is no evidence concerning the association between femoral stretch test and crossed SLR and long-term outcome. For Naffziger sign evidence is limited and shows no evidence of an association with short-term outcome of global improvement.

### Non-organic signs

#### Non-organic signs – short-term outcome

Three studies reported on non-organic signs: one high quality study [37] and two low quality studies [29,73].

##### High quality studies

The high quality study found no association between non-organic signs and disability [37]. The study was set in military medical centers and included a mixed population.

##### Low quality studies

One low quality study including chronic secondary care patients found non-organic signs to be associated with poor outcome of return to work [73]. Another low quality study found inappropriate illness behaviour to be associated with overall improvement but not with being symptom-free in a mixed primary care population [29].

The mixed results could not be explained by looking at setting, duration of symptoms, or method of outcome measurement. It is unknown whether a larger sample size could have changed conclusions from the high quality study [37] and thus explain the variability of results.

#### Non-organic signs – long-term outcome

Seven studies reported on the association between non-organic signs and long-term outcome, one of high quality [52], four of moderate quality [30,41,53,58], and two of low quality [29,71].

##### High quality studies

The high quality study, confirmatory in design, included a mixed secondary care population and showed an association between non-organic signs and poor outcome of return to work [52]. This association was retained in a multivariable analysis.

##### Low and moderate quality studies

Three moderate quality studies found an association between non-organic signs and poor outcome of return to work [41,53,58]. Two of these did multivariable analyses in which the associations were not retained [53,58]. One confirmatory study also investigated use of health care services and found that patients with non-organic signs received more physical therapy and more CT scans than patients without the signs. They found no association with six other treatment modalities/diagnostic tests [41]. One study of low quality included chronic secondary care patients and found no association with return to work [71], another included acute primary care patients and found no association with a combination outcome of pain and disability [29], and one moderate quality study on acute primary care patients found no association with disability [30]. None of these studies had a clear description of the signs or a clear definition of what constituted a positive test.

The studies reporting non-organic signs to be associated with outcome were in general of higher quality and were all set in secondary care. One study included acute patients [41], one study included acute/subacute patients [58], one study included a mixed population [52], and one study included a chronic population [53].

#### Summary of for non-organic signs

There is conflicting evidence for non-organic signs being predictive of short-term outcome, however, the high quality study showed no association. There is consistent evidence of non-organic signs being predictive of long-term poor outcome of return to work. Two studies reported that having at least three out of five positive non-organic signs increased the risk of non-return to work by 18% and 19% [52,53], whereas the risk was observed to *decrease* by 7% in one study [71]. In terms of time to return to work, those with three or more non-organic signs were observed to return on average 44 days later than those with fewer signs [41]. No estimate was reported in the three remaining studies [29,30,58]. Evidence is conflicting for the other outcomes. Sample size and method of outcome measurement did not explain this variation. The studies reporting non-organic signs to be associated with long-term outcome were in general of higher quality and were all set in secondary care.

### Functional tests and leg length discrepancy

#### Functional tests and leg length discrepancy – short-term outcome

One low quality study reported on the test “attempt to sit up from supine (+/− pain)” and did not find it associated with global improvement [29]. This study failed to report on setting and duration of symptoms.

One low quality study including a mixed primary care population reported on leg length discrepancy and found no association with outcome [68], and another low quality study failed to report on it [61].

#### Functional tests – long-term outcome

Six papers reported on functional tests, three of moderate quality [27,30,31] and three of low quality [29,61,62]. Two studies reported on lifting capacity [31,62], two on sit up test [29,30], one on time in seconds for getting into and getting out of a high bed [27], and one on trouble moving during examination [61].

Four studies found no association between functional tests and long-term outcome [27,30,31,62]. One of these included acute patients in primary care [30], and three included chronic patients in secondary care [27,31,62]. One low quality study found the sit up test in combination with other clinical tests to be associated with the outcome of being symptom-free but not the outcome of improving [29]. One low quality study found an association between trouble moving during examination and “having a difficult course” and use of health care services or medication but failed to report on disability [61]. Both of these studies were set in primary care and included mixed populations.

#### Leg length discrepancy – long-term outcome

One study of low quality on chronic patients found no association between leg length discrepancy and return to work [64], and one study failed to report on the test [61].

#### Summary of functional tests and leg length discrepancy

For functional tests evidence is limited and shows no evidence of an association with long-term outcome. We found no evidence concerning short-term prognostic value of functional tests or short-term or long-term prognostic value of leg length discrepancy.

## Discussion

We systematically reviewed the literature on clinical examination findings as prognostic factors published between 1977 and June 2012 and found that this field has been investigated only unsystematically. For example, the most thorougly studied test, the SLR test, was investigated in five high quality studies using four different definitions of a positive test and four different outcome measures. We found that symptom response classification (centralization) was the only factor with consistent evidence of an association with short-term recovery of pain (conflicting for disability), and non-organic signs was the only factor associated with long-term outcome of return to work (conflicting for other outcomes). Four tests did consistently not demonstrate an association with short-term outcome: palpation for pain, tone or symmetry; spinal ROM; SI-pain provocation tests; and muscle endurance. Similarly, there was consistently no evidence of an association with long-term outcome for four factors : palpation for pain, tone or symmetry; spinal ROM; pain on spinal ROM; and neurological signs. For all other clinical tests, evidence of any association with outcome was either limited, conflicting or non-existent.

### Comparing our results to previous literature

Our findings are in line with a review by Chorti et al. dealing with the prognostic value of symptom response in the conservative management of spinal pain which found strong evidence for an association between symptom response and both pain and work status, but no association with number of days on sick leave and inconclusive evidence for an association with disability and use of health care services [17]. In that review there was no differentiation between short- and long-term follow-up, and in several of the included studies, patients were treated according to directional preference which was an exclusion criteria in our review because a prognostic effect could not be separated from a treatment effect.

Our findings in relation to non-organic signs are similar to results in a review by Valat et al. [74] and a review by Chou et al. [75]. However, these finding are in contrast to results in a review by Kent et al. in which a significant association between non-organic signs and long-term outcome of participation restriction was found in only one of six included studies [18].

In line with previous reviews [19,76], we found no evidence for neurological signs being predictors of long-term outcome (evidence was conflicting in relation to short-term outcome), and that evidence was conflicting concerning SLR as a predictor of outcome [18,19,76]. Finally we found no associations between spinal ROM and short- or long-term outcome. Authors of two earlier reviews on LBP prognosis have found conflicting evidence of the prognostic value of spinal ROM [18,19], however in one review, very few studies on the test were included [18], and in the other, only studies dealing with chronic LBP were reviewed [19].

Although the results for individual tests in this review are discouraging, they might have prognostic value when combined. Grouping patients according to results of combinations of several clinical tests and assessment of pain was investigated in a pilot study classifying primary care patients into 10 diagnostic classes based on a clinical examination, which found that membership of a diagnoctic class at baseline was associated with the total number of days with LBP over three months [76].

Physical findings may potentially improve prediction of outcome when combined with psychological and social factors. The biopsychosocial model of back pain has become the dominant model in conceptualization of the etiology and prognosis of back pain; adressing psychological and social factors are recommended by clinical guidelines [10]; and psychological factors are consistently associated with the prognosis of patients with LBP [77,78], although not sufficiently strong to predict outcome in individuals [18]. The challenge may be to develop reliable and valid composite test batteries or instruments that are based on known biological, psychological and social risk factors. Such instruments could serve the dual purpose of estimating prognosis and stratifying patients into paths of care that optimize their chance of a good outcome [79].

Lastly, clarity about which baseline characteristics are prognostic factors and which are potential treatment effect modifiers may help outline the best management strategy. Some factors might predict outcome regardless of treatment, whereas some are only related to the response to specific treatments. Evidence exists that some factors predict treatment response but not overall prognosis [38,39,46]. Further knowledge of treatment effect modifiers may help optimize treatment effects which, for commonly used interventions, are small [80-82].

### Strengths and limitations

Due to its comprehensiveness and its detailed description of findings, this review enables a complete overview of the extent, type and quality of the research dealing with the association between all reported clinical tests and outcome in LBP patients. The confidence in our conclusions, however, is limited by the methodological shortcomings and lack of reporting clarity in many of the included papers. We were often unable to extract all of the relevant information from the reports, and where this is the case, it is recommended the original investigators be contacted [83]. We did not do so because many of the articles with missing information dated so far back, that we judged it impossible to retrieve the information. Drawing firm conclusions on the basis of this evidence is further complicated by the fact that most studies had a considerable risk of bias, and even for the most commonly studied tests, the number of studies investigating the tests in association with each outcome was limited. Furthermore, studies investigated the tests in different patient populations and various settings, employing different treatment methods, and using a broad range of definitions of tests and a great variation in definitions of outcome that were often measured in non-standardized ways and with different timing of follow-ups. Not only did the outcomes differ, but the same outcome could be treated differently in different studies, for example, a continous scale might be evaluated in its original form in one study and dichotomized with an arbitrary cutpoint in another, potentially yielding different results. However, our results showed no signs of a systematic difference on this basis. The large heterogeneity of studies and frequent lack of reporting the strength of alleged associations prevented us from providing measures of effect sizes. Moreover, one fourth of the studies were nested in randomized trials with highly selected patient samples, and also for some of the cohort studies it was often unclear whether they were truly representative of the source populations. We should thus be cautious in generalizing the findings. We recognize that conclusions based on statistical significance should be made with caution, but there was no reason to suspect that potentially important associations were missed solely because studies were not adequately powered.

Finally, searching prognosis literature can be challenging as relevant studies are often poorly indexed [84]. Studies reporting on the prognostic value of physical examination findings are often designed for other purposes, for example randomized clinical trials where the prognostic value of the clinical tests is a secondary objective of the study. Therefore, we may have missed relevant literature. Furthermore, selection bias may have been introduced as we only included studies in English, Danish, Swedish and Norwegian.

## Conclusions

Reports of the prognostic value of clinical examination findings are numerous but most studies are not designed with the primary purpose of evaluating the prognostic ability of the examination. In addition, the overall quality of the studies is low to moderate. To make progress in the area, studies need to be designed specifically to investigate the prognostic value of the clinical tests and the performance and interpretation of the tests have to be standardized. There is evidence from confirmatory studies for an association between centralization and non-organic signs and outcome. For all other tests, included studies are explorative and show either no evidence for or large uncertainty about the prognostic value of the tests.

However, most clinical tests are designed and used for other purposes, and a poor association with prognosis does not discredit the test as being diagnostic or otherwise informative for clinical management. Clinical tests may still have potential as treatment effect modifiers or as part of comprehensive predictive models.

## Additional files

Additional file 1:
**Pubmed search strategy.**
Additional file 2:
**Coding taxonomy for predictor variables.**
Additional file 3:
**Definitions of outcome variables as used in studies.**
Additional file 4:
**Descriptive table.**
