# Supplementary material for: Clinical examination findings as prognostic factors in low back pain: a systematic review of the literature
Source: Chiropr Man Therap. 2015 Mar 23;23:13. doi: 10.1186/s12998-015-0054-y (PMC4369880; doi:10.1186/s12998-015-0054-y)
Supplement: Additional file 1: — Pubmed search strategy. [file 12998_2015_54_MOESM1_ESM.docx]

**Additional file 1: Pubmed search strategy**

1. Population

Back pain OR low back pain OR intervertebral disc displacement OR sciatica OR piriformis syndrome OR sacroiliac joint syndrome OR “Facet syndrome” OR “derangement syndrome” OR “postural syndrome” OR “dysfunction syndrome” OR low back dysfunction OR lumbopelvic pain OR (referred pain AND (spine OR spinal OR back)) OR (strains and sprains AND (spine OR spinal OR back)) OR ((leg pain) AND (spine OR spinal OR back))

1. Prognosis, course and study design

Prognosis OR predict* OR cohort study OR prospective study OR case-control study OR longitudinal study OR clinical trial OR randomized controlled trial

1. 1 AND 2
2. Clinical examination/individual tests

Physical examination OR diagnostic tests, routine OR clinical examination OR clinical indicators OR physical measures OR “objective findings” OR Mckenzie classification OR clinical predictors OR clinical prediction rule OR treatment effect modifiers OR “abdominal strength” OR abnormal sensation OR “applied kinesiology” OR “back strength” OR Biering-Sorensen test OR centralisation OR centralization OR extension OR Fabere OR femoral nerve test OR flexion OR fingertip-to-floor test OR Gaenslen test OR “Gillet test” OR hip range of motion OR hypermobility OR hypomobility OR Janda OR knee extension OR Laseque OR “lateral flexion” OR leg length inequality OR Schober OR “motion palpation” OR “muscle spasm” OR “muscle strength” OR “muscle tightness” OR “neurological signs” OR “pain provocation tests” OR “palpatory tenderness” OR “pelvic compression test” OR pelvic separation test OR posture OR prone knee bend OR “prone instability test” OR psoas tightness OR range of motion, articular OR “repeated movement” OR rotation OR sacral thrust OR sacroiliac joint asymmetry OR sacroiliac joint movement OR “segmental mobility” OR side-shift OR “slump test” OR “spinal palpation” OR spinal percussion OR straight leg raise OR strength assessment OR “symptom response” OR tendon reflex OR thigh thrust OR Trendelenburg OR trigger points OR trunk breadth OR trunk strength OR “Waddell´s signs”

1. 3 AND 4
2. limit 5 to (human and English or Danish or Swedish or Norwegian)
3. limit 6 to adults 19+ years
