# Supplementary material for: Clinical examination findings as prognostic factors in low back pain: a systematic review of the literature
Source: Chiropr Man Therap. 2015 Mar 23;23:13. doi: 10.1186/s12998-015-0054-y (PMC4369880; doi:10.1186/s12998-015-0054-y)
Supplement: Additional file 2: — Coding taxonomy for predictor variables. [file 12998_2015_54_MOESM2_ESM.docx]

**Additional file 2: Coding taxonomy for predictor variables**

*Grouping of tests*

| Symptom response classification | Centralization  Peripheralization |
| --- | --- |
| Range of motion (ROM) | Spinal ROM  Aberrant movement on spinal ROM  Pain on spinal ROM  FFD  Modified Schober  ROM of the hip  Sacroiliac joint (SI) motion symmetry tests |
| Palpation | Palpation for tone, pain or asymmetry  Palpation for mobility  SI palpation |
| Pain provocation tests | Kempfs test  SI provocation tests  Prone instability test  Percussion |
| Muscle strength and endurance | Muscle strength (not neurological)  Muscle endurance |
| Neurological tests | Neurological signs  Cross SLR  Femoral stretch  SLR  Naffziger sign |
| Nonorganic signs | Nonorganic signs |
| Functional tests | Functional tests  Leg length discrepancy |

*Variations covered under each individual test (descriptions as used in studies)*

| **Symptom response classification** | Centralization | |
| --- | --- | --- |
|  | Centralization with single movement testing | |
|  | Periferalization | |
|  | Periferalization with single movement testing | |
| **Range of motion (ROM)** |  | |
| Spinal ROM | T12 and S1-2 extension, flexion, lateral flexion | |
|  | Limited sagittal lumbar mobility | |
|  | Limited passive lumbar movement | |
|  | Extension | |
|  | Flexion | |
|  | Ease of flexion | |
|  | Flexion in degrees (4 categories) | |
|  | Flexion (poor to low, good, unknown) | |
|  | Limitation in amplitude of movement | |
|  | Lateral flexion | |
|  | Flexion and extension | |
|  | Rotation | |
|  | Thoracolumbar rotation | |
|  | Trunk flexibility assessed by sit and reach test | |
| Aberrant movement on spinal ROM | Instability catch | |
|  | Painful arc of motion | |
|  | Thigh climbing | |
|  | Reversal of lumbopelvic rhytm | |
| Pain on spinal ROM | Pain-related restriction of mobility | |
|  | Pain on extension | |
|  | Pain on standing extension | |
|  | Pain on supine extension | |
|  | Pain on flexion | |
|  | Pain on lateral flexion | |
|  | Pain on rotation | |
|  | Number of painful movements | |
| Fingertip to floor distance (FFD) | Greater FFD | |
|  | FFD > 24 cm | |
|  | FFD ≥ 17 cm | |
| Modified Schober | Modified Schober | |
|  | Schober | |
| ROM of the hip | Flexion | |
|  | Extension | |
|  | Abduction | |
|  | Rotation | |
| Sacroiliac (SI) motion symmetry tests | Standing flexion | |
|  | Seated flexion | |
|  | Long-sitting | |
|  | Prone knee bend | |
|  | Gillet | |
| **Palpation** |  | |
| Palpation for tone, pain or asymmetry | Palpation – deep | |
|  | Finger pressure on paraspinal area elicited radicular pain  Pain on palpation | |
|  | Tenderness of muscles | |
|  | Tenderness of 6 points in low back and legs | |
|  | Paraspinal muscle tone | |
|  | Paraspinal muscle spasm | |
|  | Palpation of six bony landmarks for asymmetry | |
|  | Sacral sulcus palpation test | |
|  | Valleix points | |
|  | Ligamentous laxity on a 9-point scale (higher number indication more laxity) | |
|  | Number of painful spots in shoulder/neck area | |
|  | Number of painful spots in lumbar area | |
|  | Segmental pain provocation | |
| Palpation for mobility | PA mobility for each lumbar level | |
|  | Spinal stiffness of most symptomatic level | |
|  | Segmental hypermobility | |
|  | Segmental hypomobility | |
|  | Spring test | |
| **Pain provocation tests** |  | |
| Kempfs test | Kempfs test | |
| SI provocation tests | Gainslen | |
|  | Posterior shear | |
|  | Compression/distraction | |
|  | Patrick | |
|  | Resisted hip abduction | |
|  | Sacral thrust | |
| Prone instability test | Prone instability test | |
| Percussion | Percussion | |
| **Muscle strength and endurance** |  | |
| Muscle strength (not neurological) | Static trunk muscle strength | |
|  | Dynamic trunk muscle strength | |
|  | Isokinetic muscle strength | |
|  | Maximum isometric extension | |
|  | Trunk fleksion | |
|  | Trunk extension | |
|  | Hip flexion | |
|  | Hip abduction | |
|  | Active SLR | |
|  | Active sit-up | |
|  | Back and abdomen | |
|  | Abdominals | |
|  | Arm strength | |
|  | Ability to do squats |  |
| Muscle endurance | Biering-Sorensen |  |
|  | Modified Biering-Sorensen |  |
|  | Abdominals |  |
|  | Isometric abdominal muscle endurance |  |
|  | Back |  |
|  | Isometric back flexors |  |
|  | Isometric back extensors |  |
|  | Lateral flexors (side support test) |  |
|  | Index based on sit-ups, back extensions, hip extensions |  |
| **Neurological tests** |  |  |
| Neurological signs | Nerve root tension: SLR + neurological signs  Neurological signs  2 or more: ankle and patella reflexes, sensory loss, weakness in foot and/or thigh muscles  Reflexes, strength, sensibility. Present if any of them were positive  Abnormality of reflexes, impaired reflex (ankle/patella)  L4, L5, S1  Achilles reflex  Knee reflex  Strength, motor deficit, muscle weakness  Motor changes in leg  Weakness in L5 or S1 nerve root distribution  Muscle strength: great toe extension  Muscle strength: toe-heel test  Muscle strength: toe- and heel walk  Manual strength test  Paresis  Measurable muscle atrophy  Sensibility  Sensory changes  Sensory deficit  Hypoaesthesia  Light touch (hypaesthesia)  Pain on sensation (hypalgesia)  Sensory loss in an anatomic distribution  Rhomberg´s |  |
| SLR | SLR  SLR > 75 degrees  SLR > 50 degrees  SLR - limited  SLR discrepancy  SLR sitting  SLR – pain on SLR  SLR – left and right typical sciatica  Laseque´s sign (positive Laseque = evoked radiating pain in leg beyond the knee)  Bragard |  |
| Cross SLR | Cross SLR |  |
|  | Reversed Laseque |  |
| Femoral stretch test | Femoral stretch test |  |
| Naffziger sign | Naffziger sign |  |
| **Non-organic signs** | Non-organic signs: 1 or > of 8  3:5 positive  ≥ 3 signs  Simulated axial loading  Simulated rotation  General overreaction to examination/disproportionate verbalization  Superficial tenderness  Regional weakness  Widespread nonanatomic pain  Regional sensory deficit  Distracted SLR  Pain on cough/sneeze  Signs of inappropriate illness behaviour  Behavioural signs:  ”Two out of four”  The step test  The pseudo strength test  ”Sham” sciatic tension test (SLR with plantarflexion of foot) |  |
| **Functional tests** | Lifting ability  Jumping  Pushing and pulling  Lumbar lifting ability (lift from floor to waist)  Cervical lifting ability (lift from waist to shoulder)  Time to complete walk  Trouble moving during examination |  |
| Leg length discrepancy | Leg length discrepancy |  |
