# Supplementary material for: Clinical examination findings as prognostic factors in low back pain: a systematic review of the literature
Source: Chiropr Man Therap. 2015 Mar 23;23:13. doi: 10.1186/s12998-015-0054-y (PMC4369880; doi:10.1186/s12998-015-0054-y)
Supplement: Additional file 3: — Definitions of outcome variables as used in studies. [file 12998_2015_54_MOESM3_ESM.docx]

**Additional file 3: Definitions of outcome variables as used in studies**

| **PAIN** | **References** |
| --- | --- |
| Leg pain change score | 25 |
| Back pain, leg pain | 27,67 |
| LBP rating scale | 32, |
| VAS 0-10, VAS 0-100 | 35,51,59 |
| 4-point Likert scale (mild to very severe pain) | 34 |
| Pain frequency on 5 point-scale | 35 |
| 0-10 - average 24 hours | 36 |
| Reduction of at least 50% in VAS lasting beyond the first month after treatment | 42 |
| Average pain last week 0-10 NRS | 44,43 |
| Pain reduction versus no pain reduction or even higher levels of pain | 47 |
| LBP free = maximum pain score of 1 of 10 | 54 |
| Pain | 56 |
| Time to recovery from the index episode af LBP. The episode was considered to have lasted until the start of the first 4 week pain-free period | 70 |
| 6 point rating scale | 63 |
| Recurrence of back pain | 63 |
| LBP Rating Scale | 67 |
| Duration of initial period | 28 |
| The summarized duration of recurrences of pain | 28 |

Abbreviations: LBP: Low back pain, VAS: Visual analog scale, NRS: Numeric rating rcale

| **Disability** | **References** |
| --- | --- |
| RMDQ | 25,32,36,44,43 |
| Recovered if ≤ 4 on RMDQ at both 4weeks and 3 months follow-up | 44,43 |
| RMDQ: Recovered: Score of 0-2, not recovered: Score >2 | 30 |
| Oswestry Disability Questionaire | 35,38 |
| Patient specific functional status, RMDQ | 36 |
| Oswestry Disability Questionaire – Success: ≥ 50% improvement | 37,39,40 |
| Oswestry Disability Questionaire – Success: ≥ 50% improvement OR improvement <50% but >6 points. Failure: < 6 points | 46 |
| Absence of disability (maximum Oswestry score of 15 of 100) | 54 |
| ODQ > 10 | 66 |
| ODQ: Good and poor responders.  Poor responders = deterioration or no change in ODQ | 48 |
| ODQ: Lifting capacity | 55 |
| Change in level of activities of daily living | 27,56 |
| Good outcome, bad outcome | 57 |
| Bedrest | 61 |
| 0-24 scale. High score= 14/24 | 63 |
| Perceived disability | 67 |

Abbreviations: RMDQ: Roland Morris Disability Questionaire, ODQ: Oswestry Disability Questionaire

| **Return to work (RTW)** | **References** |
| --- | --- |
| RTW | 33 |
| Ability to work, disability pension obtained or application pending | 27 |
| Work retention: Number of days at work during the 2-year follow-up period. Failure = 3 days off in a row due to LBP or 5 days within a 12 months period | 31 |
| Receiving no social transfer payments other than unemployment payment in the 52nd week after inclusion – register based | 32 |
| Time to return to regular work without restrictions | 41 |
| Sickness absence | 43,64 |
| Back-to-work vs not working | 47,60 |
| Returners (returned to work) and non-returners  (remained on sick-leave) | 49 |
| RTW and the duration of time off work between beginning of treatment and RTW | 50 |
| Returned in any capacity | 52 |
| RTW: Improvement in actual work activity. Non-return: Patients without improvement and patients on vocational measures | 53 |
| Working or not | 55,64 |
| Cumulative number of days a claimant received benefits for one year from the date of the accident | 58 |
| Complete RTW(100%); incomplete RTW(<100%) | 71 |
| Success: RTW or work status improvement  (within 12 weeks considered treatment related) | 73 |
| Days absent from work. Time period not described | 63,67 |
| Recurrences. Chronicity ≥ 25% of days during one year on sick leave | 66 |
| Duration of sick-leave during the initial episode | 28 |
| Number of sick leave days since last questionaire | 65 |
| Number of new sick leave periods | 66 |
| Total absence from work owing to recurrences | 28 |
| *RTW:* ”Success group”: Completed program and back to work at one year FU. ”Failure” group: Completed program but not back to work. Drop-out group: Dropped out of the program before completing  ”Failed to enter” group: Did not enter after initial evaluation. | 62 |

| **Use of Health Care services and medication** | **References** |
| --- | --- |
| Medical resources used (8 different measures) | 41 |
| Use of health care and use of medication | 43 |
| Medication | 61 |
| Use of provider | 56 |
| Radical treatment (nucleolysis or discectomy) if inadequate response to at least two months of conservative care | 69 |
| Surgery, medication | 67 |

| **Global Improvement** | **References** |
| --- | --- |
| Global improvement based on patient’s and clinician’s opinion | 26 |
| Self rated overall assessment | 27 |
| Patients rating of back situation as much worse, worse, unchanged, better, or much better | 36 |
| Worsened, unchanged, improved, improved greatly. Major improvement after 2 weeks defined as major improvement as reported by the patient. Poor outcome after 3 months was defined as absence of improvement or eventual surgery. | 72 |
| Somatic and depressive distress (Zung + Medicare secondary payer questionaire + combining) | 35 |
| Patients subjective rating of success | 47 |
| Patients subjective opinion of efficacy | 68 |

| **Combination outcome** | **References** |
| --- | --- |
| Symptom free and improving (both composite pain and disability) | 29 |
| *Complicated* versus *light* course  *Complicated* defined as being on sick leave for > 30 days or use of pain medication > 99 days or being bedridden for > 10 days | 61 |
| Three categories - *Poor outcome*: On sick leave between questionaires or not functionally recovered at follow up. *Fair outcome*: Functionally but not completely recovered (able to do ordinary daily activities but not well-being with regard to LBP) with no sick leave at any time between questionaires. Good outcome: No indication of LBP at any time between questionaires | 65 |
| Recovery = no pain (VAS) or disability (RMDQ) | 33 |
| Recovered if Pain = 0 (VAS 0-10 cm) and disability score < 4 (RMDQ) | 45 |
