# Supplementary material for: Clinical examination findings as prognostic factors in low back pain: a systematic review of the literature
Source: Chiropr Man Therap. 2015 Mar 23;23:13. doi: 10.1186/s12998-015-0054-y (PMC4369880; doi:10.1186/s12998-015-0054-y)
Supplement: Additional file 4: — Descriptive table. [file 12998_2015_54_MOESM4_ESM.docx]

**Additional file 4: Descriptive table**

| First author  Year of publication Country | Study design | Follow-up periods | Baseline sample size and response rates at each point of FU | Setting and subjects | Description of LBP | Type of treatment | Outcome measures | Predictor variables (tests) | Results  Univariat  [multivariate] |
| --- | --- | --- | --- | --- | --- | --- | --- | --- | --- |
| Albert et al  (2012), Denmark^25^ | Prospective cohort study  Secondary analysis of data from RCT | 8 w  12 m | 176/181/?**  8 w:  165 (94%)  12 m: ? | Consecutive ptt referred to specialist spine center | Radicular pain of dermatomal distribution  ≥ 3 on NRS  2w-1y | Symptom-guided (MDT) or sham exercises for  8 w  4-8 treatments | P: Leg pain change score  D: RMDQ change score | 9. centralization  a. centralization  b. perifeheralization  c. no effect | 9.  [+] D c vs. a/b  [+] P c vs. a/b |
| Amundsen et al  (2000), Norway^26^ | RCT + longitudinal cohort | 6 m  1 y  4 y  10 y | 68/?  6 m, 1 y: ?  4 y: 64 (94%)  10 y: 48 (71%) | Consecutive ptt referred to hospital department of neurology  50 selected for conservative care  18 randomized for conservative care | Leg pain +/- back pain + radiologic signs of stenosis and compression of the clinically afflicted nerve root(s)  Not dics bulge or herniation | 20/68 surgery + orthosis + 1m in hospital rehabilitation  48/68 1m in hospital orthosis + back school + instruction. Physical training after discharge | GI: Based on patient’s and clinician’s opinion – tool unknown | 6.SLR | 6. - |
| Bendix et al  (1998), Denmark^27^ | RCT | 1 y | 816/816  Intervention  BL: 621  1 y: 534 (86%)  Control:  BL: 195  1 y: 157 (81) | Ptt referred to Copenhagen Back Center | Disabling back pain > 6 m  Threatened job status | Functional restoration  program  Control groups of no treatment or less intensive programs | P: Back pain, leg pain  D: Change in level of activities of daily living  RTW: Ability to work  Disability pension obtained or application pending  GI: Self rated overall assessment | 13.Muscle endurance  a. Isometric abdominal muscle endurance  b. Isometric back muscle endurance  15. Functional tests  a. Mobility (time in sec. for entering and leaving a high bed) | 13.  a. -  b. [+] RTW (pension), [+] pain , [-] ability to work,  [-] D, [-] GI  15. - |
| Bergquist-Ullman et al  (1977), Sweden^28^ | RCT | 1y | 217/?  1 y: ? | Health-centers at Volvo factories | LBP ≤ 3m  A pain-free y before onset of the current episode | 1. Back School  2. Combined physiotherapy  3.Placebo (shortwaves) | P: Duration of initial period  The summarized duration of recurrences of pain  RTW: Duration of sick-leave during the initial episode  Total absence from work owing to recurrences | 2. Modified Schober  3. ROM  a. Extension  b. Lateral flexion  c. Rotation  6. SLR  8. Neurological signs  (sensibility, strength of great toe extension, patella and achilles  reflexes)  12. Muscle strength  a. Sit-ups  b. Back muscle strength | 2. -  3. -  6. -  8. -  12. - |
| Burton et al  (1991), England^29^ | Prospective cohort study | 1m  3m  1 y | 109/113  1 m: 99 (91%)  3 m: 87 (80%)  1 y: 89 (82%) | Sequential ptt attending orthopaedic out-patient clinic and and office practice | LBP +/- leg pain  Mixed duration | Conservative care (manipulation, injection, advice, exercise, medication) | Composite outcome P+D: Symptom free, improving | 3.ROM spine  a. Flexion  b. Extension  c. Flexion+extension  4.ROM hip  a. Passive resisted hip flexion (prone)  b. Passive flexion of both knees  c. Passive flexion/adduktion of hip (supine)  6.SLR <50 degrees  8.Neurological signs  a. Sensory changes  b. Motor changes in leg  c. Nerve root tension tests  14.Non-organic signs  (signs of inappropriate illness behavior)  15.Functional tests  a. Attempt to sit up from supine (+/- pain) | Symptom free/improving*  3.  a. 1 m:[-]/[+], 3 m:[-]/[+],  1y: [-]/[-]  b. 1 m:[+]/[-], 3 m:[-]/[-],  1 y: [-]/[+]  c. 1 m:[-]/[-], 3 m:[-]/[-],  1 y: [-]/[-]  4.  a. 1 m:[-]/[-], 3 m:[-]/[-],  1 y: [+]/[-]  b. 1 m:[-]/[-], 3 m:[-]/[-],  1 y: [-]/[-]  c. 1 m:[-]/[-], 3 m:[-]/[-],  1 y: [+]/[-]  6. 1 m:[-]/[-], 3 m:[+]/[-],  1 y: [+]/[+]  8.  a. 1 m:[-]/[-], 3 m:[-]/[-],  1 y: [-]/[-]  b. 1 m:[-]/[-], 3 m:[-]/[-],  1 y: [-]/[-]  c. 1 m:[+]/[-], 3 m:[-]/[-],  1 y: [+]/[-]  14. 1 m:[-]/[+], 3 m:[-]/[+],  1 y: [-]/[-]  15.  a. 1 m:[-]/[+], 3 m:[-]/[-],  1 y: [+]/[-] |
| Burton et al  (1995), England^30^ | Prospective cohort study | 1 y | 252/?  1 y: 186 (74%) | Consecutive ptt consulting group practice of osteopaths | New episode of LBP | Manipulative therapy + exercise and general advice when appropriate  Average 6.6 treatments | D: RMDQ  Recovered: RMDQ score of 0-2  Not recovered:  RMDQ score >2 | 3. ROM  6. SLR  8. Neurological signs:  Root tension yes/no  14. Non-organic signs:  Overt pain behavior, 5 point scale  15. Functional tests  a. Sit-up test, 3 point scale | 3. [-]*  6. [+]*  8. [+]*  14. [-]*  15. [-]* |
| Campello et al (2006), USA^31^ | Observational prospective cohort study | 2 y | 67/71  2 y: 100% | Consecutive ptt referred to hospital-based outpatient clinic | NSLBP +/- leg pain to above knee level  Off duty or on restricted duty for >8 w and had to be receiving compensation for a work-related back injury prior to program  Participants were excluded if they did not RTW after completion of the program | Multidisciplinary work-conditioning program, 4h/d , 5d/w for 4w | RTW: Number of d that the subject remained at work during the 2 y FU period  Failure = 3 d off in a row due to LBP or 5 d within a 12 m period. Information from insurance company | 3.ROM spine  a. Flexion  b. Extension  4. ROM hip  a. Flexion  b. Extension  c. Abduction  12.Muscle strenght (not neurological)  a. Hip flexion  b. Hip abduction  c. Lower and upper abdominal  13. Muscle endurance:  Biering-Sorensen method  15. Functional tests  a. Lifting capacity | 3. -  4. -  12. -  13. -  15. - |
| Christiansen et al (2010), Denmark^32^ | Prospective cohort study nested in RCT | 1 y | 331/351  1 y P+D:  235/331= 71%    1 y RTW: 330/331 (100%) | Ptt referred from GPs to outpatient Spine Center | LBP +/- sciatica  Sick-listed  36,9% had nerve root pain  50% > 3 m duration | Brief intervention versus multidisciplinary intervention | P: LBP rating scale  D: RMDQ  RTW: Register based; defined as receiving no social transfer payments other than unemployment payment in the 52nd week after inclusion | 9.  a. Centralization  b. Peripheralization  c. No response | 9.  a. -[-]  b. -[-] |
| Coste et al  (1994), France^33^ | Prospective  cohort study | Time to event  1-7 d diary  8 d visit  15 d visit  30 d visit  60 d visit  90 d visit | 103/?  FU: 94 (89% )  Ptt not followed after registration of recovery | Consecutive ptt self referring to GPs (39 practices) | Acute (< 72 h) localized NSBP (not below glut fold). | Paracetamol  Bed rest and/or sick leave at the discretion of GPs | Recovery: No P (VAS 0-10) or D (RMDQ)  RTW: Not defined | 3. ROM spine  a. Limited passive lumbar movement  b. Aberrant movement (catch)  6.SLR <75**°** | 3  a. - recovery, - RTW  b. - recovery, +[-] RTW  6. - recovery, - RTW |
| Dwornik et al (2007), Poland^34^ | Prospective cohort study | 3 w | 50/?  3 w: 50 | ? | Conditions of the back of the trunk > 3w duration  ICD10 codes: M40-M54.9 + G50 – G59.8 | Non-specific physiotherapy (laser, cryo therapy, electrodes, massage, kinesiotherapy) | P: 4-point Likert (mild to very severe pain) | 2. Schober  3. ROM spine  6. SLR  a. SLR  b. Bragard  c. Reversed Laseque  7.Cross SLR  8.Neurological signs  a. Reflexes  b. Sensation  c. Toe-heel test  10.Palpation  a. Tenderness 6 points low back + legs  b. Paraspinal muscle tone | 2. ?  3. ?  6. ?  7. +  8. ?  10. ? |
| Enthoven et al (2003), Sweden^35^ | Prospective descriptive study | 12 m | 55  12 m:  44 (80%) | 2 primary health care centers (GPs and PTs) | LBP that could be provoked by combined side flexion, ipsilateral rotation and extension, or sustained maximal flexion  Mixed duration | No specific treatment (advice and medication) | P: VAS 0-100,  Pain frequency on 5 point-scale,  D: ODQ  GI: Somatic and depressive distress (Zung + Medicare secondary payer questionaire + combining) | 1. FFD  3. ROM spine  Thoracolumbar rotation  6.SLR  13.Muscle endurance  a. Isometric back flexors  b. Isometric back extensors | 1. -  3. -  6. -  13.  a. + P, - D  b. - |
| Ferreira et al (2009), Australia^36^ | Prospective cohort study nested in RCT | 8 w | 191/240/?  8 w: ? | 3 hospital based outpatient physiotherapy departments | NSLBP for ≤ 3 m | Spinal manipulative therapy, motor control exercise, or a general exercise program  12 sessions in 8 w | P: 0-10, average 24 h  D: Pt specific functional status, RMDQ.  GI: Global perceived effect 11 point Likert | 10. Palpation  Spinal stiffness of most symptomatic level | 10. [-] for all OM |
| Flynn et al (2002), USA^37^ | Prospective cohort study | 3^rd^ treatment | 75/?  3^rd^ treatment:  71 (95%) | Military medical centers  Ptt referred for physiotherapy | NSLBP  Pain or numbness in the lumbar spine +/- legs  ODQ ≥ 30%  Mean duration 41.7 d | Manipulation + simple exercise + advice to keep active | D: Success = ≥ 50% reduction in ODQ score | 3.ROM lumbar spine  4.Hip rotation  5.SI-tests  SI motion symmetry tests  a. Standing flexion  b. Seated flexion  c. Long-sitting  d. Prone knee bend  e. Gillet  SI provocation tests  a. Gaenslen  b. Posterior shear  c. Compression/  distraction  d. Patrick  e. Resisted hip abduction  f. Sacral sulcus palpation test  g. Sacral thrust  Palpation of bony landmarks for asymmetry (6)  6.SLR  9.Centralization/  perifeheralization with single movement testing  10. Palpation  a. Segmental hypomobility  b. Segmental pain provocation  14.Non-organic signs | 3. -  4. + left, -[+] right  5.  SI motion tests  a. -  b. -  c. -  d. -  e. -  SI provocation  a. -  b. –  c. -  d. -  e. -  f. -  g. -  - Palpation of bony landmarks for asymmetry  6. -  9. -  10.  a +[+]  b. -  14. - |
| Fritz et al (2004), USA^40^ | Prospective cohort study  Based on same cohort as Flynn (2002) | 3^rd^ treatment | 75/?  3^rd^ treatment:  71 (95%) | Military medical centers  Ptt referred for physiotherapy | NSLBP only  Pain or numbness in the lumbar spine +/- legs  ODQ ≥ 30%  Mean duration 41.7 d | Manipulation + simple exercise + advice keep active | D: Success = ≥ 50% reduction in ODQ score | 3. ROM lumbar spine  4. Hip rotation  5. SI-tests:  SI motion symmetry tests  a. Standing flexion  b. Seated flexion  c. Long-sitting  d. Prone knee bend  e. Gillet  SI provocation tests  a. Gaenslen  b. Posterior shear  c. Compression/distration  d. Patrick  e. Resisted hip abduction  f. Sacral sulcus test  g. Sacral thrust  Palpation of bony landmarks for asymmetry (6)  6. SLR  9. Centralization/ perifeheralization with single movement testing  10.  a. Segmental hypomobility  b. Segmental pain provocation  14. Non-organic signs | 3. -  4. + left, -[+] right  5.  SI motion tests  a. -  b. -  c. -  d. -  e: -  SI provocation  a. +[+]  b. -  c. -  d. -  e. -  f. -  g. -  - Palpation of bony landmarks for asymmetry (+ for pubic tubercle asymmetry in supine)  6. -  9. -  10  a +[+]  b. -  14. - |
| Fritz et al (2007), USA^38^ | RCT | 2 w  6 w | 64/?  2 + 6 w:  49 (77%) | Ptt at 4 outpatient physiotherapy clinics | LPB + signs of nerve root compression in past 24 h  ODQ >30%  Median duration 47,5 d | 6 w of extension-oriented intervention +/- mechanical traction during the first 2 w | D: ODQ | 3.ROM spine  a. Flexion  b. Extension  6. SLR  7. Cross SLR  9.  a. Centralization  b. Periferalization  10. Palpation  a. Segmental hypermobility  b. Segmental hypomobility | 3. -  6. -  7. -  9.  a. +  b. –  10. - |
| Fritz et al (2005), USA^39^ | Prospective cohort from RCT | 4 w | 131/157  4 w:  125 (95%) | 2 academic medical centers; 6 outpatient practices  Most facilities within the Air Force | LBP + ODQ ≥ 30%  No clinical signs of nerve root compression  Median duration 27 d | Manipulation/  stabilization exercise  or stabilization exercise alone | D: Modified ODQ  Success: ≥ 50% improvement | 10. Palpation  a. PA segmental hypomobility  b. PA segmental hypermobility | 10.  a. -  b. - |
| Gaines et al  (1999), USA^41^ | Consecutive case series | FU every 7-10 d until RTW  (Range:  2-219 d) | 55/55  FU: 100% | Consecutive ptt visiting directly or referred to multispecialty clinic | Acute work-related LBP without radicular signs  Acute defined as LBP for the first time in at least 1 year and now present for < 10 w | Education, medication, modified RTW assignment, 4-6 physiotherapy visits | RTW:  Time to return to regular work without restrictions  UHC:  Medical resources used (8 different measures) | 14.Non-organic signs (≥ 1 of 8)  a. Simulated axial loading  b. Simulated rotation  c. General overreaction to examination  d. Superficial tenderness  e. Reagional weakness  f. Widespread, nonanatomic pain  g. Regional sensory deficit  h. Distracted SLR | 14.  +[?] RTW,  +[?] UHC 2:8 measures,  - UHC 6:8 measures  a. + RTW, ? UHC  b. + RTW, ? UHC |
| Ghahreman et al (2011), Australia^42^ | Prospective study based on RCT | 1 m | 79/?  1 m: 71 (90%) | Consecutive ptt seen by neurosurgeon at hospital  6 were inpatients of the hospital and 65 were outpatients | Lumbar radicular pain caused by CT verified  disc herniation  SLR < 45 degrees  65/71 > 6 w duration | Transforaminal injection of steroids | P: VAS  Favorable response defined as a reduction of ≥  50% in VAS lasting beyond the first m after treatment | 8. Neurological signs  a. Sensory deficit  b. Abnormality of reflex  c. Motor deficit | 8. - |
| Grotle et al  (2005), Norway^44^ | Inception cohort study | 4 w  3 m | 123/?  4 w, 3 m:  120 (98%) | Ptt consulting primary care for the first time: 43% GPs, 25% Chiropractors, 5% PTs, 27% recruited through advertisement | LBP +/- radiation Duration < 3 w | Treatment as usual in primary care | P: Average pain last week 0-10 on NRS  D: RMDQ  RTW: Sickness absence  Recovery: Recovered if ≤ 4 on RMDQ at both 4 w and 3 m FU | 1.FFD  3.ROM spine (sidebending)  8.Neurological signs (2 or more)  a. Ankle and patella reflexes  b. Sensory loss  c. Weakness in foot and/or thigh muscles  d. SLR | 1.? P, ? D, -[-] recovery  3. ? P, ? D, ? recovery  8. + P, + D, +[+] recovery |
| Grotle et al  (2007), Norway^43^ | Inception cohort study  Based on same cohort as Grotle 2005 | 1 y | 123/?  1 y: 112 (91%) | Ptt consulting primary care for the first time: 43% GPs, 25% Chiropractors, 5% PTs, 27% recruited through advertisement | LBP +/- radiation Duration < 3 w | Treatment as usual in primary care | P: Average pain last week 0-10 on NRS  D: RMDQ  RTW: Sickness absence  Secondary outsomes:  UHC + use of medication  Non-recovery:  > 4 on RMDQ | 1. FFD  3.ROM spine (FFD sidebending)  8.Neurological signs (2 or more)  a. Ankle and patella reflexes  b. Sensory loss  c. Weakness in foot and/or thigh muscles  d. SLR  e. Radiation into foot | 1. ? P, ? D, ? RTW,  ? UHC, -[-] non-recovery  3. ? P, ? D, ? RTW,  ? UHC, ? non-recovery  8: +[-] P, +[-] D, ? RTW,  ? UHC, -[-]non-recovery |
| Gurcay et al  (2009), Turkey^45^ | Prospective  study | 2 w  12w | B: 99/?  2+12 w:  91 (92%) | Consecutive ptt at hospital outpatient clinic (tertiary referral and training center)  Blue- and white collar workers  with insurance | Acute LBP < 3w  No neurological deficits | Medication on as-needed basis  Short time bed rest | Combination outcome:  Recovered if Pain = 0 (VAS 0-10 cm) and disability score < 4 (RMDQ) | 1. FFD  6. SLR  8. Neurological signs  a. Impaired ankle/patella reflex  b. Sensory loss  c. Muscle weakness  11.Paravertebral muscle spasm | 1. +[-]  6. -[-]  8. ?  11. -[-] |
| Hicks et al  (2005), USA^46^ | Prospective cohort study | 8 w | 57/?  8 w: 57 (95%) | 3 outpatient PT clinics and 1 outpatient clinic at airforce base | LBP +/- leg pain  Maximum one neurological sign  Mixed duration (mean 40.6 ± 44.2 d) | Stabilization program twice weekly for 8 w + daily home exercises | D: ODQ  Success: ≥ 50% improvement  Improvement: <50% but > 6 points on ODQ  Failure: < 6 points on ODQ | 3A. ROM lumbar  3B. Aberrant movement  a. Instability catch  b. Painful arc of motion  c. Thigh climbing  d. Reversal of lumbopelvic rhythm  5.SI-test (posterior shear test)  6. SLR  10. Palpation  a. PA lumbar segmental mobility  b. Ligamentous laxity on a 9-point scale (higher number indicating more laxity)  12. Muscle strenght  a. Active sit-up  b. Active SLR  13. Muscle endurance  a. Modified Biering-Sorensen  b. Lateral flexors (side support test)  18. Other (prone instability test) | 3A. -  3B.  +[+] success, +[+] failure  5. -  6. -[+] success, - failure  10.  a. - success, +[+] failure  b. -  12. -  13. -  18.  +[+] success, +[+] failure |
| Hildebrandt et al (1997), Germany^47^ | Prospective cohort study | 8 w  6 m  12 m | ?/?  8 w: 90 (?%)  6 m: ?  12 m:  82 (91%) | Hospital department  81% received full compensation  30% had prior back surgery  68% showed signs of depression  48% reported non-specific bodily pain  Standard treatment had failed | Chronic back pain  At least 3 m off work during past y  26% had radicular pain | Rehab. program: Multidisciplinary treatment of functional restoration | P: Pain reduction versus no pain  RTW: back-to-work versus not working at discharge  GI: Patients rating of success | 1. FFD  3. ROM | 8 w*:  1. ?[-] P, ?[-] RTW,  ?[+] GI  3. ?[-] P, ?[-] RTW,  +[-] GI  6+12 m*: ? |
| Hurri et al  (1989), ? ^48^ | RCT | 12 m | 204/?  12 m:  177 (87%) | ? | LBP ≥ 1y  Symptoms during month preceding initial exam | Treatment group: Education, exercise 6 times in 3 w + 2 review classes 6 m later  Control group: Handout. Free to use the health care services they were used to | D: ODQ  Good and poor responders  Poor responders = deterioration or no change in ODQ score | 3. ROM spine  a. Flexion  b. Lateral flexion  10. Palpation  a. Number of painful spots in the lumbar area  b. Number of painful spots in the shoulder-neck area  12.Muscle strenght  a. Dynamic trunk muscle strength  b. Static trunk muscle strength  c. Ability to do squats | 3*.  a. +  b. +  10*.  a. +  b. +  12*.  a. +  b. +  c. + |
| Indahl et al  (1998), Norway^49^ | Prospective cohort  One treatment arm from controlled trial, unclear if randomised | 5 y | 245/245  5 y:  245 (100%) | All ptt referred to hospital spine clinic | LBP of 4-12 w duration | “Mini Back School” (pt education) | RTW:  Returners = ptt that returned back to work  Nonreturners = ptt that remained on sick-leave | 1. FFD  12. Isokinetic muscle strength | 1. +[-]  12. ? |
| Infante-Rivard et al  (1996), Canada^50^ | Prospective clinical trial | Returners: 4-1127d  Non-returners: 293-1228d  Retired,  went into vocational training or education: 69-880d  Lost to FU:  14-892 d | 305/402 (76%)  FU: 270 (89%) | Workers with first compensated episode of LBP referred to one of two rehabilitation centers approved by health insurance | LBP  No sick leave due to LBP for last 5 y  Duration unclear | Conventional therapy (assage, heat, exercises, lumbar traction, etc.) Discharge decided by treating physician | RTW: RTW and duration of time off work between beginning of treatment and RTW | 3. ROM  a. Flexion  b. Limitation in amplitude of movement  8.Neurological signs (reflexes, strength and sensibility. Present if any of them were positive) | 3.  a. +[+]  b. -  8. -[-] |
| Jamison et al (1991), USA^51^ | Prospective cohort study | 2 w | 249/?  2 w: 249 | Ptt referred to hospital-based pain center  Randomly selected by order of admission  Failed conservative treatment  No psychiatric disorders | LBP + radicular symptoms  Duration ≥ 3 m, mean 2.7 y | Lumbar epidural steroid injection | P: VAS | 6.SLR  8.Neurological signs  a. Sensory  b. Motor | 6. -  8. - |
| Karas et al  (1997), Canada^52^ | Observational cohort study | 6 m | 126/154  6 m:  126 (82%) | Consecutive ptt referred to Canadian Back Institute rehabilitation clinic  Working population | LBP +/- leg pain without signs of neurological impairment  Duration 2 w-2 y | Active exercise regardless of centralization status or Waddell score  1-3 h/d for 30 d  Home exercises at discharge | RTW: Returned in any capacity. Based on blinded telephone interviews | 14. Non-organic signs (≥3 signs) | 14. +[+] |
| Kool et al (2002), Switzerland^53^ | Prospective cohort study | 12 m | 99/?  12 m:  90 (91%) | Ptt referred from physicians to rehabilitation clinic  Off work due to LBP > 6 w within previous 6 m | CLBP | Rehabilitation (exercise, training, back school)  Average stay in rehabilitation center 28 d | RTW: Improvement in actual work activity  Non-return: Ptt without improvement and ptt on vocational measures  Data obtained from treating physician | 14. Non-organic signs  (≥ 3:5 signs) | 14. +[-] |
| Leboeuf-Yde et al  (2004), Norway^54^ | Prospective cohort study | 4th visit  3 m  12 m | 875/?  4th visit:  799 (91%)  3 m:  598 (68%)  12 m:  875 (58%) | Consecutive ptt from 115 chiropractors each including about 10 ptt  No treatment by a chiropractor during preceding 6 m | Pain T12 -lower gluteal folds  Duration ≥14 d  Pain ≥ 30 d in total during preceding 12 m | Choice of treatment up to each chiropractor (manipulation,  information, massage, traction, exercise,  advice) | P: LBP free = maximum  ≤ 1 of 10  D: Absence of disability = maximum ODQ score ≤ 15 of 100  Measured at 4th visit, 3 and 12 m | 3.ROM (pain)  a. Pain on flexion  b. Pain on extension  c. Pain on lateral flexion  d. Pain of rotation  e. Number of painful movements  10. Palpation  (pain on palpation) | 3. -  10. - |
| Long et al  (1995), Canada^55^ | Prospective comparative cohort | Discharge  (1-18 w)  9 m  (3-18 m)  2 y  (2-3 y)  P  discharge  D: discharge + 2 y  RTW:  9 m + 2 y | 223/243 (92%)  Discharge: ?  9 m: 166 (74%)  2 y: 53% Unknown whether the response rate is based on BL or FU | Consecutive ptt at  privately owned interdisciplinary rehabilitation facility  All ptt were receiving compensation | CLBP +/- leg symptoms | Work-hardening program (physiotherapy, exercise conditioning, work simulation, education, psychological intervention)  Average duration 5 d/w for 11 w | P: NRS 0-100  Minimum, maximum, average pain)  D: ODQ: Lifting capacity  RTW: Working or not | 9. Centralization/non-centralization | 9.  Discharge: + P, - D  9m:  - D, + RTW  12m:  - D, - RTW |
| Lonnberg  (2010), Denmark^56^ | Prospective cohort study | 22 y | 78/?  22 y: 47 (60%) | Consecutive ptt in GP practice seeking care for the first time because of LBP  61% >3 prior episodes | LBP +/- leg pain  Pain from L3 to S1/iliac creast, between lateral borders of quadratus lumborum muscles  Mixed duration (19% > 4 w) | Usual GP care. | P: Tool not described  D: Limitations to daily living  UHC: Use of provider | 3. ROM spine (pain-related restriction of mobility)  6. SLR | 3. -  6. - |
| Luoto et al  (1998), Finland^57^ | Prospective cohort study | 6 m | 68/?  6 m: 65 (96%) | Consecutive ptt  at rehabilitation center | Moderate CLBP that caused trouble in work and everyday life | Back rehabilitation program of active functional restoration, 3 w in-patient, 2x3 d pre- and post- course | D: Good outcome = decreased disability  Bad outcome = no change or increased disability | 13. Muscle endurance  a. Static back endurance  b. Squatting (repetitions) | 13.  a. -  b. - |
| McIntosh et al (2000), Canada^58^ | Prospective cohort study | 1 year | 2007  1 y:  1752 (87%) | Ambulatory rehabilitation facilities  Claimants with acute or subacute LBP who received lost-time benefits for a work injury | Acute or subacute LBP | ≤ 30 d of exercise 1-3 h/d | RTW:  Cumulative number of d a claimant received benefits for 1 y from the date of the accident | 6.SLR  8. Neurological signs (L4, L5, S1)  14. Nonorganic signs  18. Femoral nerve stretch | 6. -  8. + S1, - L4, L5, [-] L4, L5, S1  14. +[-]  18. - |
| Michaelson et al (2004), Sweden^59^ | Prospective cohort study | 4 w  12 m | 315 Neck pain ptt and LBP ptt  4 w:  303 (96%)  LBP:  4 w: 167 (?%)  12 m:  129 (?%) | Consecutive ptt at inpatient rehabilitation center | LBP > 6 m  Pain ≥ 25 mm/100mm VAS | Multimodal treatment (physical + cognitive-behavioral) 6 h/d,  5 d/w, 4 w | P: VAS | 13. Muscle endurance  (index based on sit-ups, back extensions, hip extensions) | 13: - |
| Milhous et al (1989)  USA^60^ | Prospective cohort study | 6 m | 87/? | Ptt admitted to an orthopedic back clinic  All unemployed at time of study  28 had had surgery | LBP  Mixed duration | No treatment | RTW: Returned to work or not | 6. SLR  8. Neurological signs (leg strength, sensation and reflexes) | 6. -  8. - |
| Pedersen  (1980), Denmark^61^ | Prospective observational cohort study | 1 m  3 m  6 m  1 y | 78/83  1 m: 95%  3 m: 95%  6 m: 95%  1 y: 92%  Unknown whether response rate based on BL or FU | All ptt consulting GP clinic for the first time due to first episode of LPB within a year. | NSLPB  Pain from L3 to S1/iliac creast, between lateral borders of quadratus lumborum muscles  50% + leg pain, 20% distal to the knee  Mixed duration | Usual care | D. Bed rest  UHC: Medication  Combination outcome:  *Complicated* versus *light* course  *Complicated* defined as being on sick leave for > 30 d or use of pain medication > 99 d or being bedridden for > 10 d | 6.SLR  8.Neurological signs  a. Paresis  b. Reflexes  18.  a. Trouble moving (during examination)  b. Leg length discrepancy | 6. + D, ? UHC, ? C  8. ?  18.  a. ? D, + UHC, + C,  b. ? |
| Polatin et al  (1989), USA^62^ | Prospective cohort study | 1 y | 326/?  1 y: 246 (75%) | A medical center and a rehabilitation institute | CLBP ptt considered candidates for functional restoration program | Functional restoration program | RTW:  *Success group* (125): Completed program and back to work at 1 y  *Failure group* (121): Completed program but not back to work  *Drop-out group* (40): Dropped out of the program before completing  *Failed to enter group* (40): Did not enter after initial evaluation | 3. ROM  a. True lumbar flexion and extension  b. True flexion  c. True extension  4. ROM hip  a. Hip flexion  15. Functional tests  a. PILE lifting (Progressive Isoinertial Lifting Evaluation) | 3.  a. -  b. +[?]  c. -  4.  a. +[?]  15.  a. - |
| Roland (1983), England^63^ | Prospective cohort study | 1 w  1 m  (P, D, RTW)  1 y Recurrence | 215/?  (215 ptt, 230 episodes)  1 m: 181 episodes (79%)  1 y:  201 ptt (94%) | GP group practice | LPB +/- leg pain  No consultation for LBP in preceding 28 days | Usual GP care  (in 94% only prescription of simple analgesics) | P: 6 point scale  Recurrence of pain  D: 0-24 scale  High score= 14/24  RTW: Days absent | 3. ROM spine (pain or limitation)  a. Flexion  b. Extension.  c. Lat. Flexion  4. ROM hip (rotation)  5. SI-test (pain on straining anterior and posterior SI-ligaments  6. SLR  a. SLR > 60 degrees  b. Back pain on dorsi-flexion of foot at maximum SLR  7. Cross SLR  8. Neurological signs  a. Knee+ankle reflexes  b. Muscle strength  c. Abnormal neurological signs | 3. ?  4. ?  5. ?  6.  a. ? P ,+ D, ? RTW  b. ? P, + D, + RTW,  7. ?  8.  a. ? P, ? D, ? RTW  b. ? P, ? D, ? RTW  c. ? P, ? D, + RTW,  1 y (recurrence of pain):  3., 4., 5., 6., 7. -  8a. + |
| Sandström et al (1986), Sweden^64^ | Prospective cohort study | 1 y  2 y | 52/52  1 y: 50 (96%)  2 y: 100%? | Consecutive ptt referred to department of orthopaedic surgery | CLBP for ≥ 3m  Sick-listed  No neurological disturbances | Individually adjusted rehabilitation | RTW:  1 y: Working and non-working  2 y: Sickness absence from registries | 3. ROM spine  6. SLR  8. Neurological signs  a. Sensation  b. Reflexes  c. Weakness of leg ml.  d. Atrophy of leg ml.  e. Babinski´s reflex  10. Muscle spasm  a. Increased tonus of paraspinal muscles  18. Other (leg length) | 1 y:  3. -  6. -  8. -  10. -  18. -  2y: ? for all |
| Schiottz-Christensen et al (1999), Denmark^65^ | Prospective cohort study | 1 m  6 m  1 y | 524/?  1 m:  509 (97%)  6+12m:  503 (96%) | 75 GPs | LBP < 14 d duration  No episodes in previous 6 m | Usual care | Poor outcome:  On sick leave or not able to manage ordinary activity  RTW:  Number of sick leave days since last questionnaire | 3. ROM spine  (restriction yes/no)  6. SLR (radiating pain on SLR <60 degrees)  8. Neurological signs  a. Missing reflexes in the leg (yes/no)  b. Muscular paresis in the leg (yes/no)  16. Percussion test | Poor outcome 6 and 12 m  3. -  6. -  8.  a. -  b. -  16. -  RTW 1 m:  6. +[-]  3., 8., 16. ? |
| Seferlis et al (2000), Sweden^66^ | Prospective cohort study nested in RCT | 12 m | 180/?  12 m:  D: 123 (68%)  RTW:  174 (97%) | Ptt referred from GPs, occupational therapists or emergency department | Acute LBP +/- leg pain  Sick leave for < 2 w Employed  No treatment within last month | Manual therapy or  intensive training  or usual GP care  (= control group) | D: ODQ (>10)  RTW:  a. Number of new sick leave periods  b. Chronicity ≥ 25% of d on sick leave during 1 y  (from social insurance office) | 3. ROM (sagittal mobility)  6. SLR | 3. -  6. - |
| Skytte et al  (2005), Denmark^67^ | Prospective cohort study | 1 m  2 m  3 m  6 m  12 m | 60/60  1 m: 90%  2 m: 88%  3 m: 93%  6 m: 95%  12 m: 92%  Unknown whether the response rate is based on BL or FU | Consecutive ptt referred from primary care to rheumatology department at university hospital for suspected disc herniation | Back pain + leg pain and sciatica  Duration < 14 w | Routine structure including advice, exercise and analgesics | P : LBPRS  Back pain and leg pain.  D: Perceived disability  RTW: Days on sick leave  UHC: Surgery, medication | 9.Centralization/non-centralization | 9.  1 m: - P, + D, - RTW  2 m: + P(leg), - P(back),  + D, - RTW  3 m: + P, + D, - RTW  6 m: - P, - D, - RTW  12 m: - P, - D, - RTW  + UHC (surgery)  All FU: - UHC  (medication) |
| Sweetman et al (1996), England^68^ | Prospective study nested in RCT | Short term? | 301/?  FU: 301 (?%) | ? | LBP | 4 treatment groups:  Shortwave diathermy, traction, exercise and control (sub-thermal diathermy) | GI: Patients subjective opinion of efficacy (EFFS) | 1. FFD  a. FFD  b. FFD mobile (able to reach floor)  3.ROM spine  a. Limited sagittal mobility  b. Pain on standing extension  c. Pain on supine extension  d. Pain on flexion  e. Painfull end point on flexion  f. Pain on lateral flexion  4.ROM hip (rotation)  6.SLR  a. Limited SLR  b. Pain on SLR  8.Neurological signs  a. Hypoaesthesia  b. Knee/ankel reflexes  c. Weakness in L5 or S1 nerve root distribution  d. Pain on femoral stretch  10. Palpation  a. Spinous process springing pain  b. Ilio lumbar angle tenderness on pressing  18. Other (unequal leg length) | 1.  a. +  b. +  3.  a. -  b. -  c. +  d. -  e. +  f. -  4. -  6.  a. +  b. +  8.  a. +  b. +  c. +  d. -  10.  a. -  b. -  18. - |
| Valls et al (2001), France^69^ | Prospective study | 11-24 m  mean FU 18 m | 140/140  FU: 134 (96%) | Consecutive ptt admittet to hospital rheumatology department for conservative care | Disc-related LBP + sciatica  Mixed duration  >3/6 criteria of Saporta and/or confirmatory imaging | Complete bedrest, intravenous ketoprofen infusions for six days + if needed epidural bethamethasone injection on alternate days. Relative rest and use of lumbar support for 1 m recommended at discharge | UHC: Radical treatment (nucleolysis or discectomy) if inadequate response to at least 2 m of conservative care. | 2. Schober  3. ROM spine  6. SLR  a. SLR pain  b. SLR degrees  7. Cross SLR  a. Cross SLR pain  b. Cross SLR degrees  8. Neurological signs  a. Strength  b. Sensation  c. Reflexes  10.Palpation  a. Finger pressure on paraspinal area elicited the radicular pain | 2. -  3. +[-]  6.  a. +[+]  b. -  7.  a. ?  b -  8. -  10. - |
| Van den Hoogen et al (1997), Netherlands^70^ | Prospective cohort study | 1 y | 443/605 (73%)  1 y: 269 (61%) | Consecutive ptt in 11 general practices (15 GPs) | LBP or radiation from the back  Nonspecific and suspected specific LBP  Mixed duration | Usual care  GP + PT for some | P:  Time to recovery from the index episode of LBP  The episode was considered to have lasted until the start of the first 4-w pain-free period.  Occurrence of relapse: Pain in 1 or more of the w of the FU period, lasting up till next 4-w pain-free period | 2. Modified Schober  6. SLR  a. Limited SLR (< 80 degrees)  b. Laseque´s sign (radiating pain in leg beyond the knee) | 2. +[-]  6.  a. -[-]  b. -[-] |
| Vendrig et al (1999), Netherlands^71^ | Prospective study | 6 m | 143/147 (98%)  6 m:  137 (96%) | Consecutive ptt referred to multidisciplinary assessment and intervention center | LBP ≥ 3 m duration  No structural pathology | 4-week daily multimodal program with aim of restoring normal pattern of daily functioning | RTW:  Complete (100%)  Incomplete (<100%) | 14.Non-organic signs | 14. - |
| Vroomen et al (2002), Netherlands^72^ | Prospective study nested in RCT | 2 w  3 m | 183/227 (81%)  2 w:  181 (99%)  3 m:  169 (92%) | 50 GPs offices  First consultation for sciatica | First time episode of sciatica + signs of nerve root involvement  Mixed duration (median duration 16 d)  No previous surgery  No worker´s compensation claim | Bedrest or “watchful waiting” | GI: Worsened, unchanged, improved, improved greatly  Major improvement after 2 w as reported by the pt  Poor outcome after 3 m defined as absence of improvement or eventual surgery. | 1.FFD  6.SLR  8.Neurological signs  a. Paresis  b. Light touch (hypaesthesia)  c. Pain sensation (hypalgesia)  d. Reflexes  10. Palpation (Valleix points)  18.Other  a. Reversed SLR (femoral nerve stretch test)  b. Kempf sign present  c. Naffziger sign present | 1. + 2 w, - 3 m  6. - 2 w, + 3 m  8.  a. -  b. + 2 w, - 3 m  c. -  d. -  10. -  18.  a. - 2 w, + 3m  b. -  c. - |
| Werneke et al (1993), USA^73^ | Prospective cohort study | 3 m | 183/?  3 m:  170 (93%) | 2 orthopedic outpatient clinics Ptt referred from a wide variety of specialties  Average 8.7 m disability  16 had prior back surgery | CLBP  Nonworking or partially disabled | Physical reconditioning training and work simulation activities | RTW: Success = RTW or work status improvement | 14. Non-organic signs (behavioral signs scored as number of positive signs out of 8 tests | 14. + |

*Discriminant analysis. **Baseline sample size and response rates at each point of follow up: xx/xx/xx refers to included/agreed to participate/number of patients invited to participate. Xx/xx refers to included/invited. Many authors include consecutive patients but fail to report whether any patients refused to participate. Follow up rates are calculated on the basis of included patients as many of the studies fail to report on how many patients were invited to participate. Non-report of source population is dealt with in the quality assessment.

+ = statistical significant association, - = no association, ? = no result of investigated association was reported.

Abbreviations: BL: base line, h: hours, d: days, w: weeks, y: years, ptt: patients, GPs: general practitioners, PTs: physiotherapists, LBP: Low back pain, CLBP: Chronic low back pain, NSLBP: nonspecific low back pain, FU: follow up, P: pain, D: disability, RTW: Return to work, SL: Sick Leave, UHC: use of health care services, GI: Global perceived Improvement, RMDQ: Roland Morris Disability Questionaire, ODQ: Oswestry Disability Questionaire, LBPRS: low back pain rating scale, FFD: finger-floor-distance, ROM: range of motion,, SI: sacroiliac, SLR: straight leg raiser test,
